# Control of developmentally primed erythroid genes by combinatorial co-repressor actions

**DOI:** 10.1038/ncomms9893

**Published:** 2015-11-23

**Authors:** Ralph Stadhouders, Alba Cico, Tharshana Stephen, Supat Thongjuea, Petros Kolovos, H. Irem Baymaz, Xiao Yu, Jeroen Demmers, Karel Bezstarosti, Alex Maas, Vilma Barroca, Christel Kockx, Zeliha Ozgur, Wilfred van Ijcken, Marie-Laure Arcangeli, Charlotte Andrieu-Soler, Boris Lenhard, Frank Grosveld, Eric Soler

**Affiliations:** 1 Department of Cell Biology, Erasmus Medical Center, 3015CN Rotterdam, The Netherlands; 2 Inserm UMR967, CEA/DSV/iRCM, Laboratory of Molecular Hematopoiesis, Université Paris-Saclay, 92265 Fontenay-aux-Roses, France; 3 Computational Biology Unit, Bergen Center for Computational Science, N-5008 Bergen, Norway; 4 MRC Molecular Haematology Unit, Weatherall Institute of Molecular Medicine, University of Oxford, Oxford OX3 9DS, UK; 5 Department of Proteomics, Erasmus Medical Center, 3015CN Rotterdam, The Netherlands; 6 CEA/DSV/iRCM/SCSR, Université Paris-Saclay, 92265 Fontenay-aux-Roses, France; 7 Center for Biomics, Erasmus Medical Center, 3015CN Rotterdam, The Netherlands; 8 Inserm UMR967, CEA/DSV/iRCM, Laboratory of Hematopoietic and Leukemic Stem cells, Université Paris-Saclay, 92265 Fontenay-aux-Roses, France; 9 Department of Molecular Sciences, Faculty of Medicine, MRC Clinical Sciences Centre, Institute of Clinical Sciences, Imperial College London, London W12 0NN, UK; 10 Cancer Genomics Center, Erasmus Medical Center, 3015CN Rotterdam, The Netherlands; 11 Laboratory of Excellence GR-Ex, 75015 Paris, France

## Abstract

How transcription factors (TFs) cooperate within large protein complexes to allow rapid modulation of gene expression during development is still largely unknown. Here we show that the key haematopoietic LIM-domain-binding protein-1 (LDB1) TF complex contains several activator and repressor components that together maintain an erythroid-specific gene expression programme primed for rapid activation until differentiation is induced. A combination of proteomics, functional genomics and *in vivo* studies presented here identifies known and novel co-repressors, most notably the ETO2 and IRF2BP2 proteins, involved in maintaining this primed state. The ETO2–IRF2BP2 axis, interacting with the NCOR1/SMRT co-repressor complex, suppresses the expression of the vast majority of archetypical erythroid genes and pathways until its decommissioning at the onset of terminal erythroid differentiation. Our experiments demonstrate that multimeric regulatory complexes feature a dynamic interplay between activating and repressing components that determines lineage-specific gene expression and cellular differentiation.

## Abstract (web summary)

Conserved sets of transcription factors (TFs) regulate hematopoiesis. Here, Stadhouders *et al*. show that IRF2BP2 is a component of the LDB1 TF complex and together with its co-repressor ETO2, enhances transcriptional repression, which plays a crucial role at the erythroid progenitor stage.

## Graphical abstract

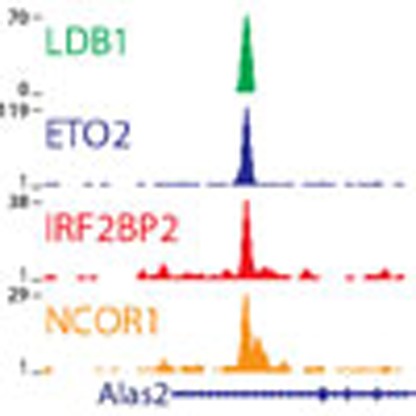

Haematopoietic development relies on the stepwise activation and repression of lineage-specific gene expression programmes. This process is regulated by sets of conserved transcription factors (TFs) acting in a combinatorial and/or antagonistic pattern to establish cellular identity through tight control of gene regulatory networks^1^. Exactly how TFs and the cofactors they recruit cooperate within large protein complexes to rapidly modulate gene expression during differentiation is still not completely understood. We set out to address this issue using a well-characterized erythroid differentiation system driven by a multimeric TF complex nucleated by the haematopoietic master regulators LIM-domain-binding protein 1 (LDB1), GATA-binding protein 1 (GATA1), T-cell acute lymphocytic leukaemia protein 1 (TAL1), LIM domain-only 2 and eight-twenty-one 2 (ETO2)—hereafter referred to as the LDB1 complex. The LDB1 complex plays a pivotal role in promoting differentiation of the erythroid and megakaryocytic lineages^2^. It was previously shown to bind the regulatory regions of developmentally regulated erythroid genes, which are rapidly induced by the LDB1 complex upon terminal erythroid differentiation^3^,^4^,^5^,^6^,^7^. Despite being already bound by the LDB1 complex in immature progenitors, premature full activation of these erythroid genes is prevented by the LDB1-complex member ETO2 (also referred to as the myeloid-transforming gene on chromosome 16 or MTG16), a transcriptional co-repressor^3^,^4^,^5^,^7^,^8^. ETO2 belongs to a family of transcriptional repressors known as the ETO family, which further consists of the founder member ETO (or MTG8) and the myeloid translocation gene, related-1 (MTGR1) proteins. ETO2 plays key roles in the maintenance of haematopoietic stem cells^9^, the development of the lymphoid system^10^ and regulating effective (stress) erythropoiesis^11^. The importance of a functional ETO2 protein in maintaining haematopoietic homeostasis is further underlined by its causal involvement in acute leukaemia^12^,^13^,^14^. Whereas ETO2 is well known for its repressor function in several cell types^3^,^15^,^16^, the molecular mechanisms of erythroid gene suppression in the context of the LDB1 complex remain largely unknown. Unravelling these mechanisms is important to provide novel insight into how TFs and cofactors within a multimeric complex impose a ‘primed' status (that is, a stage-specific transcriptional repression of late erythroid genes in immature progenitors) onto their target genes, which rapidly switches to full activation at the onset of differentiation.

In this study, to begin addressing these questions, we performed a proteomics screen for novel ETO2-binding partners. This screen identifies the interferon regulatory factor 2-binding protein 2 (IRF2BP2), growth factor-independent 1B (GFI1B) and lysine-specific demethylase 1 (LSD1) transcriptional repressors as ETO2-interacting proteins. We show here that IRF2BP2 is a novel component of the LDB1 complex able to strongly enhance ETO2-mediated transcriptional repression. Chromatin immunoprecipitation-sequencing (ChIP-Seq) analysis and loss-of-function studies reveal that ETO2 and IRF2BP2 chromatin occupancy significantly overlap at a genome-wide scale, and that both factors regulate a common set of key erythroid target genes and regulatory pathways. Subsequent analysis of IRF2BP2 protein partners shows that IRF2BP2 is able to recruit the well-known NCOR1 co-repressor, which is able to bind ETO2/IRF2BP2 erythroid target genes to potentially mediate their repression. We finally confirm the *in vivo* relevance of the newly identified IRF2BP2 co-repressor by using an IRF2BP2-deficient mouse model. Animals homozygous for the genetrap *Irf2bp2* allele display an ineffective fetal liver (FL) erythropoiesis during gestation and die around birth. Thus, our data reveal a complex collaborative action of multiple co-repressor proteins within the LDB1 complex at the erythroid progenitor stage. As a result, late erythroid-specific genes are maintained in a primed state before their rapid activation upon terminal differentiation.

## Results

### An epigenetic definition of primed LDB1 target genes

‘Primed' developmentally regulated genes have been previously defined as being already expressed at low levels before full activation at the onset of differentiation^17^. Moreover, TFs responsible for the full activation of primed genes upon terminal differentiation have been observed to already bind primed genes at the progenitor stage^18^. The late erythroid genes activated by the LDB1 complex can thus be considered ‘primed' in undifferentiated erythroid progenitors, where they are bound by the LDB1 complex but expressed at low levels^5^. To more accurately define their transcriptional and epigenetic status, we analysed the expression, TF binding, RNA polymerase II (RNAPII) and H3K4Me2 levels of primed archetypical erythroid genes in mouse erythroleukaemia (MEL) erythroid progenitors before and after the induction of terminal erythroid differentiation. RNA sequencing (RNA-Seq) analysis shows that primed erythroid genes are indeed expressed at low—but significant—levels in progenitors and are strongly (±5 fold on average) induced upon differentiation (Supplementary Fig. 1a). The vast majority (88%) is already bound by an ETO2-containing LDB1 complex in progenitors (see ref. 5). Analysis of genome-wide RNAPII ChIP-Seq data indicates that >95% of the primed genes show no overt signs of paused RNAPII accumulating at the promoter before induction, ruling out RNAPII pause-release^19^ as a general mechanism of primed gene activation (Supplementary Fig. 1b). In accordance with low-level expression and strong TF binding, the active H3K4Me2 chromatin mark is already deposited at the regulatory elements of primed erythroid genes in progenitors (Supplementary Fig. 1c). Previously published ChIP-Seq data obtained from primary erythroid progenitors further confirms this finding^20^ (illustrated in Supplementary Fig. 6e).

We conclude that primed archetypical erythroid genes in progenitor cells are uniformly defined by: (1) low expression levels that are strongly induced upon differentiation; (2) occupancy of the TFs responsible for their later induction; (3) an active chromatin environment at the regulatory elements controlling their expression and (4) not having substantial amounts of paused RNAPII at their promoters. Thus, primed erythroid genes are subjected to active transcriptional repression involving ETO2 that prevents their premature activation^5^.

### Identification of ETO2 protein partners in erythroid cells

**Figure 1:**
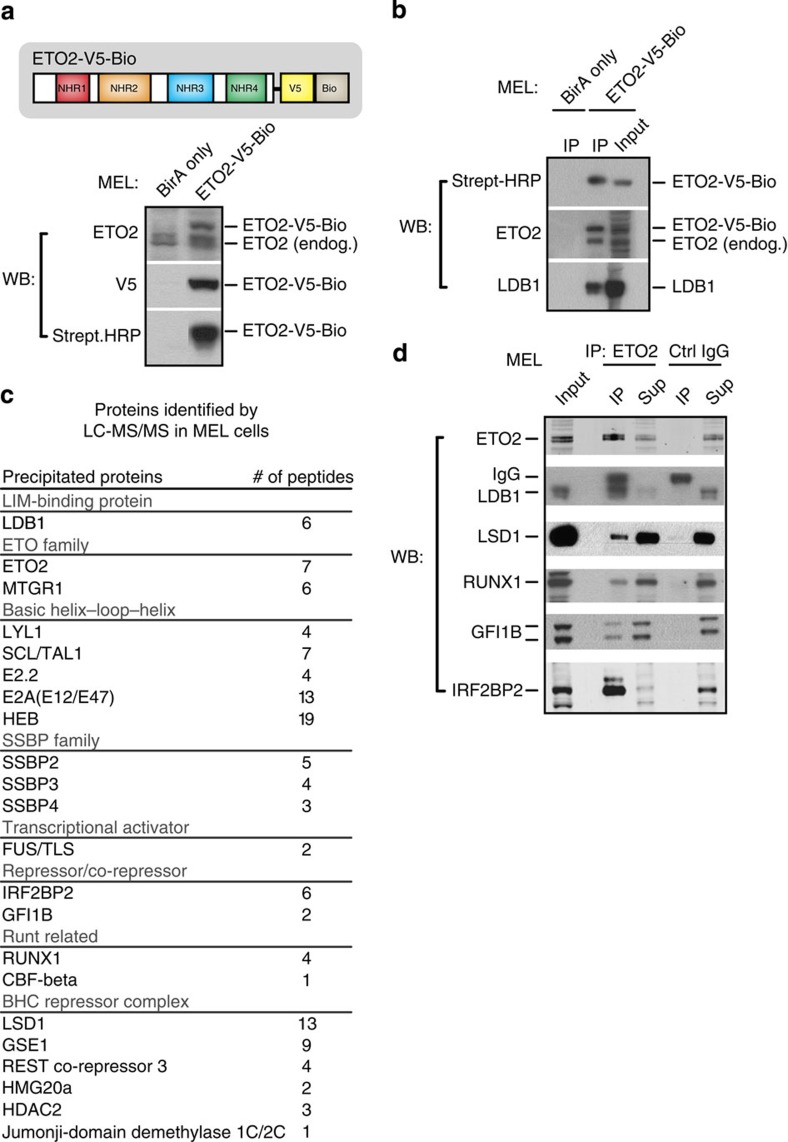
Identification of ETO2-binding partners in erythroid progenitor cells. (**a**) Schematic of the ETO2 protein, its 4 Nervy homology regions (NHR1-4) and the C-terminal V5-Bio tag (top). Fusion protein expression and proper tag function in MEL cells were validated by WB analysis. MEL cells expressing only the BirA enzyme were used as a control. (**b**) Efficient streptavidin IP of ETO2-V5-Bio in MEL cells. Interaction of ETO2-V5-Bio with LDB1 (a known binding partner) was used for validation. (**c**) ETO2-V5-Bio-interacting proteins identified by LC–MS/MS in MEL cells. Only proteins pulled down in two independent experiments and with low background scores are shown. (**d**) Co-IP validations of selected ETO2-V5-Bio-interacting proteins in MEL cells using an endogenous ETO2 antibody. Species-matched IgG was used to control for aspecific binding. Full-size images of all western blots shown can be found in Supplementary Fig. 10. Strept-HRP, streptavidin-HRP; Sup, supernatant; endog., endogenous; WB, western blot; IP, immunoprecipitation.

We next employed a proteomics approach to characterize the molecular determinants of ETO2's repressive activity. An epitope-tagged form of ETO2 (ETO2-V5-Bio) was stably expressed in MEL cells^5^ and used in single-step protein complex capture experiments^21^. The affinity tag contains a Bio peptide sequence that is efficiently biotinylated by the bacterial BirA enzyme, resulting in the biotinylation of ETO2-V5-Bio (Fig. 1a—full-size images of all western blots shown can be found in Supplementary Figs 10 and 11). The C-terminal tag fused to ETO2 does not interfere with its functions, since ETO2-V5-Bio shows (i) proper intracellular localization (Supplementary Fig. 2a), (ii) the ability to interact with its known binding partner LDB1 (ref. 7) (Fig. 1b) and (iii) binding to known genomic target sites^5^ (Supplementary Fig. 2c). Thus, tag addition does not affect ETO2 in its ability to form complexes. A streptavidin pull-down was carried out and co-purified proteins were identified by mass spectrometry (liquid chromatography–tandem mass spectrometry (LC–MS)/MS) (Fig. 1c). In addition to known components of the LDB1 complex (for example, TAL1, the E proteins E2A and HEB, single-stranded DNA-binding protein (SSBP) 2/3/4)^7^, we also detect additional interactions with the LSD1/Co-REST repressor complex, the haematopoietic TF GFI1B and the transcriptional repressor IRF2BP2. MS analysis of endogenously precipitated ETO2 protein complexes (immunoprecipitation (IP)–MS) and individual co-IP experiments in MEL cells confirm the endogenous interaction of ETO2 with these factors (Fig. 1d; Supplementary Fig. 2). Whereas the ability of ETO2 to interact with GFI1B was reported previously^3^, and the LSD1 protein was found to be associated with the LDB1 complex (including ETO2) in erythroid cells^22^, the involvement of IRF2BP2 in these complexes has not been reported yet. We therefore set out to investigate this interaction in more detail.

### ETO2 interacts with IRF2BP2 via a unique N-terminal domain

**Figure 2:**
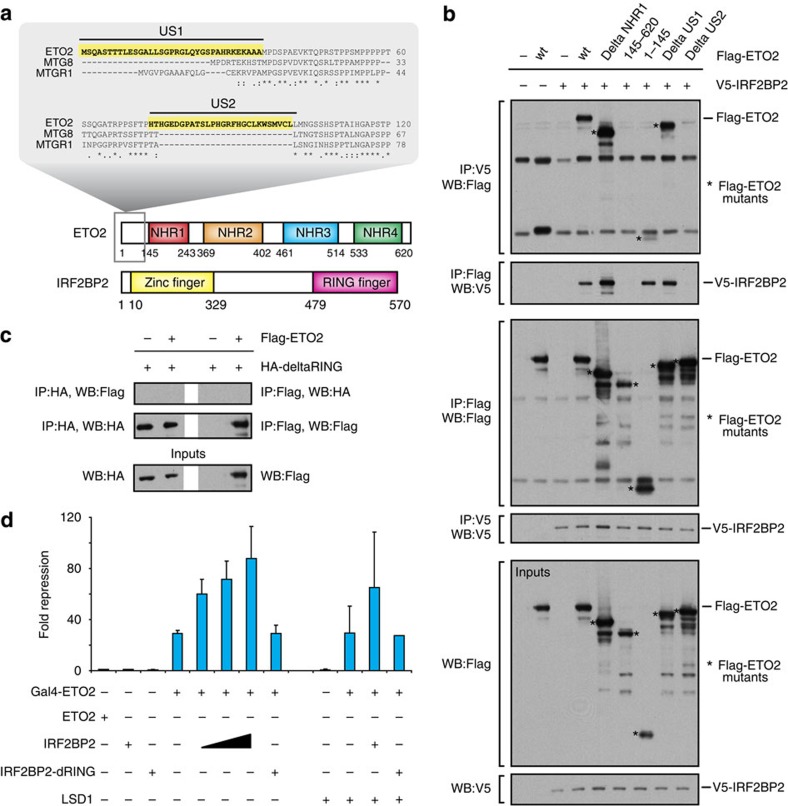
ETO2 and IRF2BP2 interact via their US2 and RING domains, respectively, to cooperatively repress reporter gene activity. (**a**) Schematic of the ETO2 and IRF2BP2 proteins and known functional domains. First and last amino-acid positions of known functional domains are indicated by numbers. Highlighted are two unique N-terminal amino-acid sequences (US1 and US2) only present in ETO2. (**b**) ETO2 interaction domain mapping using a collection of Flag-tagged deletion mutants that were overexpressed in HEK 293T cells together with V5-IRF2BP2. Bands representing the ETO2 mutant proteins are marked by an asterisk. (**c**) An HA-tagged IRF2BP2 lacking the C-terminal RING-finger domain (HA-deltaRING) was used in co-IP experiments with Flag-ETO2. (**d**) Luciferase reporter assay to test repression of a Gal4-responsive promoter (coupled to a firefly luciferase gene) by ETO2 and its interacting partners IRF2BP2 and LSD1. Fusion to a Gal4 DNA-binding domain (Gal4–ETO2) was used to target ETO2 to the promoter. Different combinations of Gal4-ETO2 and IRF2BP2, deltaRING and LSD1 were co-transfected and firefly luciferase expression was measured after 48 h. Co-transfection with equal amounts of a Renilla luciferase expression plasmid was used for normalization. Bars represent average values of at least three independent transfection experiments; error bars denote s.d. Full-size images of all WBs shown can be found in Supplementary Fig. 11. WB, western blot; IP, immunoprecipitation.

IRF2BP2 is a highly conserved zinc-finger/RING-finger protein belonging to a family of three evolutionary conserved factors (IRF2BP1, IRF2BP2 and IRF2BPL) sharing high sequence homology. IRF2BP1 and IRF2BP2 were originally identified as interacting partners of IRF2, mediating its ability to repress *in vitro* reporter expression^23^. Recently, several other studies reported a repressive role for IRF2BP2 in complex with nuclear factor of activated T-cells 1 (NFAT1)^24^, p53 (ref. 25) or enhanced at puberty 1 (EAP1)^26^, although an activating role for IRF2BP2 in regulating *VEGFA* expression has been described as well^27^. To map the domains mediating the interaction between ETO2 and IRF2BP2, a series of deletion mutants was generated and used in co-IP experiments. ETO2 contains four highly conserved domains (nervy homology regions 1–4) shared with the other members of the ETO family (ETO and MTGR1)^28^, but also two unique sequences at its N terminus not shared with ETO/MTGR1, which we termed Unique Sequence 1 and 2 (US1/2) (Fig. 2a). As shown in Fig. 2b, ETO2 interacts with IRF2BP2 via its US2 domain, suggesting that ETO2 is the only protein from the ETO family able to bind IRF2BP2. Using a similar strategy, we find that the IRF2BP2 RING-finger domain mediates the interaction with ETO2 (Fig. 2c). RING-finger domains are characteristic of E3 ubiquitin ligases catalysing the ubiquitination of target proteins, which often leads to protein degradation^29^. Since ETO2 interacts with the RING-finger domain of IRF2BP2, we tested whether ETO2 stability could be affected by this interaction. Increasing amounts of IRF2BP2 were co-expressed together with ETO2 in HEK 293T cells, and ETO2 protein levels were monitored by western blot analysis. As shown in Supplementary Fig. 3, even when expressed in large excess, IRF2BP2 does not significantly affect ETO2 protein levels under these conditions.

### IRF2BP2 enhances ETO2-mediated transcriptional repression

The functional role of the ETO2–IRF2BP2 interaction was first investigated *in vitro* using luciferase reporter assays. ETO2 was fused to a Gal4 DNA-binding domain and co-expressed in HEK 293T cells together with a luciferase reporter plasmid containing Gal4-responsive elements. As previously reported, ETO2 induces a 20–30-fold repression of luciferase activity (Fig. 2d)^30^. Co-expression of IRF2BP2 further increases ETO2-mediated transcriptional repression in a dose-dependent manner. This effect is not seen when using a RING-finger deletion mutant of IRF2BP2 (IRF2BP2deltaRING) unable to interact with ETO2. Importantly, the ETO2-interacting partner LSD1 (Fig. 1c,d), a known transcriptional repressor, does not significantly enhance ETO2-mediated repression (Fig. 2d) despite its ability to interact with ETO2 in HEK cells (Supplementary Fig. 3b).

### Genome-wide analysis of ETO2 and IRF2BP2 chromatin binding

**Figure 3:**
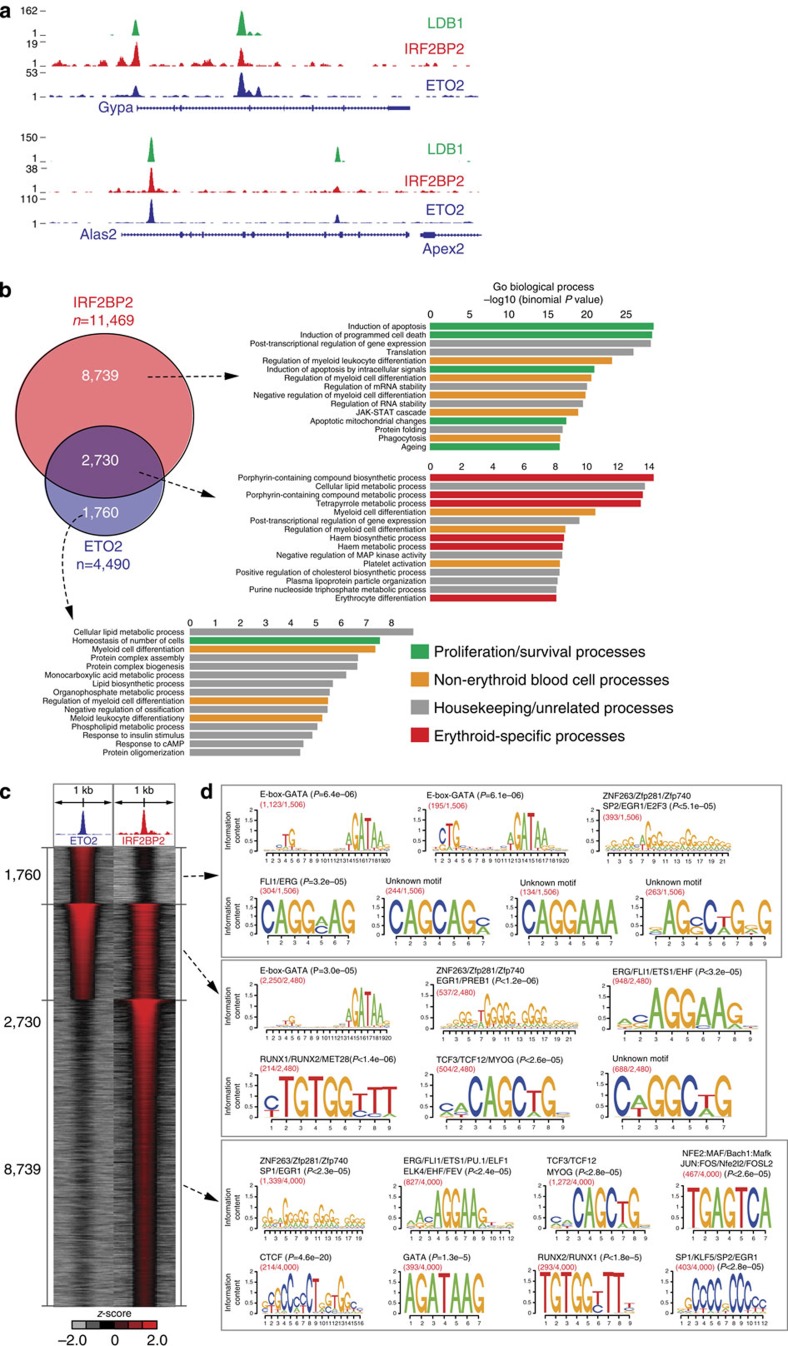
ETO2–IRF2BP2 genomic co-occupancy is associated with genes involved in key erythroid processes. (**a**) Selected examples of overlapping ChIP-Seq profiles for LDB1, IRF2BP2 and ETO2 in MEL cells on key erythroid gene loci. (**b**) Venn diagram showing the genome-wide overlap between ETO2- and IRF2BP2-binding sites in MEL cells. GREAT analysis^31^ (see Methods for more details) was performed for each group of binding sites (ETO2 only, co-occupied and IRF2BP2 only) to identify their putative target genes and possible significantly associated Gene Ontology (GO) terms. The top 15 GO terms are shown for each group of binding sites, and individual GO terms were categorized into four classes (erythroid-related, non-erythroid blood-related, proliferation/survival-related and housekeeping/unrelated). (**c**) Heatmap visualization of ETO2 and IRF2BP2 ChIP-Seq data, depicting all significant binding events centred on the peak region within a 1-kb window around the peak (binding sites were ranked on intensity). (**d**) A motif analysis (see Methods for more details) on the three groups of binding sites (ETO2 only, co-occupied and IRF2BP2 only) was performed to identify possible over-represented transcription factor-binding motifs within the peak sequences. Red numbers denote (number of motifs)/(total number of binding sites).

We next performed ChIP-Seq experiments to determine whether IRF2BP2 is enriched at critical regulatory sites occupied by ETO2. In erythroid progenitors, IRF2BP2-binding sites occur at numerous *cis*-regulatory regions of late erythroid genes controlled by ETO2 and LDB1. For example, IRF2BP2 and ETO2 show co-occupancy on the *Gypa*, *Slc22a4*, *Epb4.2*, *Alas2* and *Slc4a1* genes, as well as the α- and β-globin clusters (see Fig. 3a for examples). These genes are critical markers of mature erythroid cells and reside in a primed state in erythroid progenitors such as MEL cells^5^,^6^ (Supplementary Fig. 1). This suggests that IRF2BP2 might cooperate with ETO2 to maintain these erythroid genes in a primed state, suppressing the actions of the other LDB1-complex members required for their rapid activation upon terminal differentiation^2^,^6^. A genome-wide comparison of ETO2- and IRF2BP2-binding patterns revealed that 61% of ETO2-binding sites are also occupied by IRF2BP2 (Fig. 3b). However, many genomic locations are bound by IRF2BP2 in the absence of ETO2 and vice versa (Fig. 3c), indicating that both proteins are also involved in different regulatory complexes. Analysing peak distribution relative to transcription start sites (TSSs) (using Genomic Regions Enrichment of Annotations Tool (GREAT)^31^, see Methods) reveals that both common and ETO2-specific binding occurs predominantly (>80%) distal (>5 kb) from a TSS (Supplementary Fig. 4), in agreement with the binding distribution of the LDB1 complex^5^. However, IRF2BP2-only peaks show significantly more (42%) proximal promoter (<5 kb of the TSS) binding. Interestingly, the LSD1 and GFI1B repressors are also found enriched at ETO2/IRF2BP2-binding sites, overlapping with the positioning of the LDB1 complex (Supplementary Fig. 5).

We tried to substantiate these observations on ETO2 and IRF2BP2 chromatin occupancy by performing a Gene Ontology term analysis on putative target genes assigned to the different binding site subsets using GREAT. This confirms a strong enrichment for erythroid functions among the common target genes (Fig. 3b). ETO2-specific target genes show some enrichment for common blood-cell-related functions, as well as for several housekeeping processes. Intriguingly, IRF2BP2-specific target genes show strong associations with biological processes and functions involved in survival, apoptosis and cancer (Fig. 3b).

A *de novo* DNA motif search performed on ETO2- and IRF2BP2-occupied genomic binding sites reveals an enrichment of several different TF-binding motifs. ETS (E-twenty-six, for example, the Friend leukaemia integration 1 (FLI1)/ETS-related gene (ERG) TFs) and G/C-rich (early growth response (EGR) family) TF motifs were detected in all three categories, while Runt-related TF (RUNX) and TCF motifs show enrichment at sites occupied only by IRF2BP2 (Fig. 3d). Strikingly, nearly all ETO2-only and ETO2–IRF2BP2 shared sites contain the typical LDB1-complex signature represented by a composite E-box/GATA motif (CTGN(6–8)WGATAR)^5^,^32^, while this motif is completely absent from the IRF2BP2-only-binding sites (Fig. 3d). This shows that in MEL cells, the IRF2BP2-only-binding sites are LDB1-complex independent. However, IRF2BP2-only sites did show some enrichment for GATA motifs, indicating that a small subset of these sites could be GATA1-targeted. Interestingly, IRF2BP2-only sites are specifically enriched for several other TF motifs, including CCCTC-binding factor (CTCF), Specificity protein/Krüppel-like factor (SP/KLF) and leucine zipper (that is, activating protein-1 family) TF motifs.

### Irf2bp2 expression during erythroid differentiation

**Figure 4:**
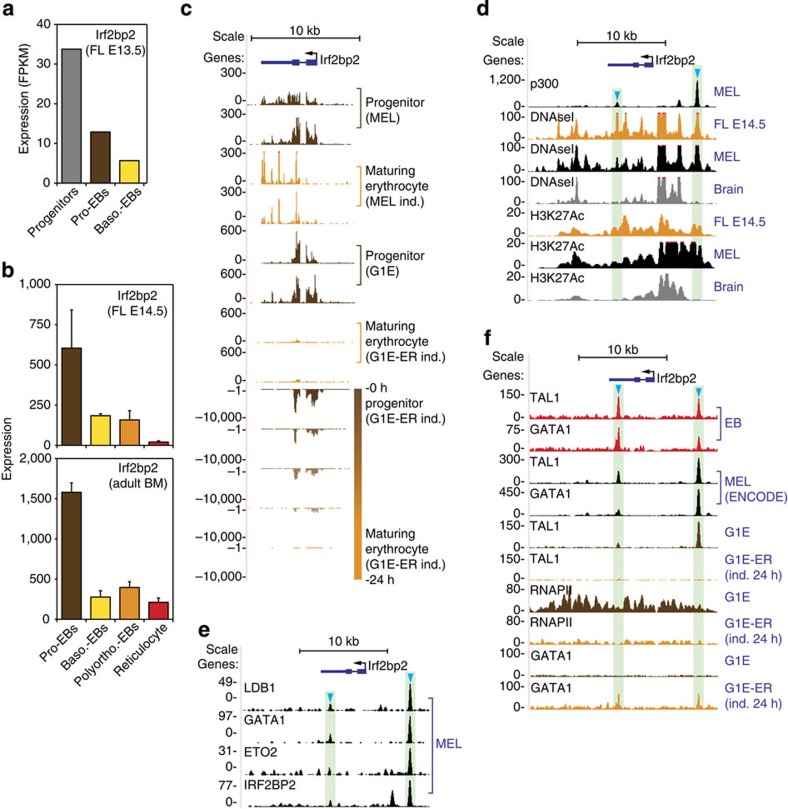
*Irf2bp2* gene expression and transcriptional regulation during erythroid development. (**a**) *Irf2bp2* expression levels at different stages of erythroid development (‘Progenitors', CD71^−^/Ter119^−^; ‘Pro-EBs', CD71^+^/Ter119^−^; ‘Baso-EBs, CD71^+^/Ter119^+^) as determined by RNA-Seq analysis of sorted E13.5 fetal liver (FL) cells (*n*=1). (**b**) *Irf2bp2* gene expression values at different stages of erythroid development in E14.5 FL and adult bone marrow (BM). Data were obtained from the online ErythronDB database^35^ (*n*=5, error bars denote s.d.). (**c**–**f**) Genome-wide data sets centred on the *Irf2bp2* locus from MEL, G1E(-ER), E14.5 FL, erythroblast (EB) and whole-brain cells. (**c**) RNA-Seq data from (differentiating) erythroid progenitors. (**d**) ChIP-Seq (p300 and H3K27Ac, both associated with enhancer activity) and DNase I-Seq (denotes regions of open chromatin) tracks; note the presence of two erythroid-specific putative enhancer elements (blue arrowheads). (**e**) LDB1 complex (including IRF2BP2) occupancy of these putative enhancer elements in MEL cells. (**f**) GATA1, TAL1 and RNAPII binding to the *Irf2bp2* locus in erythroid cells. Note the loss of TAL1 binding to the putative enhancer elements in differentiating G1E-ER cells, accompanied by a loss of RNAPII enrichments. Data shown in **c**,**d** and **f** were obtained from the ENCODE consortium^36^ (see Methods for details). Pro-EBs, pro-erythroblasts; Baso-EBs, basophilic erythroblasts; Polyortho-EBs, polyorthochromatic erythroblasts; RNAPII, RNA polymerase II.

It is well established that ETO2 expression levels diminish as erythroid progenitors undergo terminal differentiation^3^,^4^,^7^. Furthermore, in a G1E-ER model system of erythroid differentiation, expression of *Cbfa2t3* (encoding ETO2) was repressed upon GATA1-driven erythroid maturation^33^,^34^. These and other observations^34^ suggest that *Cbfa2t3* expression is regulated by the ETO2-containing LDB1 complex, which involves an ETO2 negative autoregulatory loop. To gain more insight into the regulation of *Irf2bp2* during erythropoiesis, we examined its expression levels during mouse FL erythropoiesis. RNA-Seq analysis of fluorescence-activated cell sorting (FACS)-purified populations of developing erythroid cells (the same populations as shown in Fig. 7i,j, see the Methods section for more details) indicates that *Irf2bp2* expression is reduced upon differentiation (Fig. 4a). These observations are further validated by quantitative PCR (qPCR) experiments (Supplementary Figs 8 and 9). A similar trend was observed by others using various *in vivo* and *in vitro* model systems for erythroid development^35^,^36^ (Fig. 4b,c). As was reported for *Cbfa2t3*, *Irf2bp2* expression is lost upon GATA1-driven erythroid maturation in a G1E-ER model system (Fig. 4c). In addition, using genome-wide data sets previously generated by our laboratory^5^ and the Encyclopedia Of DNA Elements (ENCODE) consortium^36^, we identify two putative enhancer elements within the *Irf2bp2* locus bound by the ETO2/IRF2BP2-containing LDB1 complex (Fig. 4d–f). When G1E-ER cells are differentiated by translocation of GATA1 into the nucleus, the TAL1 activator^2^,^5^,^32^,^37^ is displaced from these putative regulatory elements (Fig. 4f), along with a loss of *Irf2bp2* expression (Fig. 4c) and RNAPII occupancy of the locus (Fig. 4f). Collectively, these data indicate that during erythroid differentiation *Irf2bp2* expression is repressed in a GATA1-dependent manner. We speculate that, similar to events observed at the *Cbfa2t3* locus^34^, *Irf2bp2* regulation involves negative auto-regulation by ETO2/IRF2BP2.

### An IRF2BP2–ETO2 axis imposes transcriptional repression

**Figure 5:**
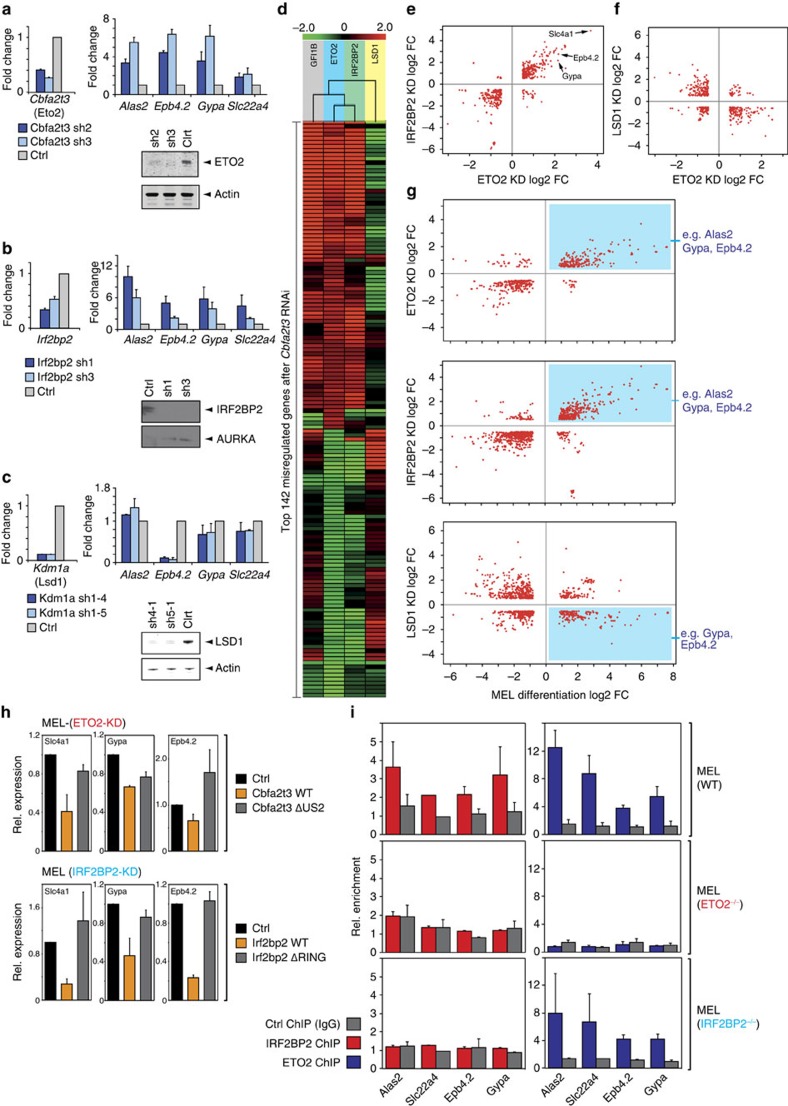
Genome-wide analysis of gene expression changes shows that ETO2 and IRF2BP2 repress the late erythroid transcriptome. Lentiviral delivery of shRNAs against *Cbfa2t3* (**a**), *Irf2bp2* (**b**) and *Kdm1a* (**c**) mRNA to deplete MEL cells of the ETO2, LSD1 and IRF2BP2 proteins, respectively. A non-targeting shRNA (Ctrl) was used as a control. After 72 h, mRNA levels were measured by qPCR (normalized versus *Rnh1* levels) or protein levels by western blot (actin and AURKA were used as loading controls). Expression levels of four archetypical late erythroid genes (*Alas2*, *Epb4.2*, *Gypa* and *Slc22a4*) were quantified by qPCR. (**d**) Unsupervised clustering of the top 142 misregulated genes after *Cbfa2t3* (ETO2) KD and the expression changes of the same set of genes induced after GFI1B, IRF2BP2 and LSD1 depletion. Gene expression changes are shown as log2 fold change (FC). (**e**–**g**) Correlations between gene expression changes (log2 FC) after ETO2/IRF2BP2/LSD1 KD (72 h post transduction) or MEL cell differentiation (96 h). Red dots represent individual genes (see Methods for more information on thresholds used). Positions of archetypical late erythroid genes (for example, *Alas2*, *Epb4.2* and *Gypa*) within the graphs are indicated. (**h**) MEL cells transduced with *Cbfa2t3*-sh3 (top) and *Irf2bp2*-sh1 (panel) lentiviruses were transfected with empty pcDNA3.1 vector (Ctrl), wild-type cDNA or with an interaction-deficient mutant *Cbfa2t3* or *Irf2bp2* cDNA (also see Fig. 2). Gene expression of three late erythroid genes was measured 48 h after transfection by qPCR (normalized versus *Rnh1* levels). (**i**) ChIP-qPCR experiments for ETO2 and IRF2BP2 in parental wild-type (WT) MEL cells, ETO2^−/−^ and BP2^−/−^ MEL cell lines generated using CRISPR/Cas9 technology. Protein binding was examined on the regulatory regions of *Alas2*, *Slc22a4*, *Epb4.2* and *Gypa*. Species-matched IgG was used as a negative control. All bars represent averages of at least two independent experiments; error bars denote s.d. Full-size images of all western blots shown can be found in Supplementary Fig. 11.

We next tried to address the functional roles played by ETO2 and IRF2BP2 in erythroid cells. Short hairpin RNA (shRNA)-mediated knockdowns (KDs) of *Cbfa2t3* and *Irf2bp2* were performed in MEL cells, after which the expression of several ETO2-LDB1 target genes was measured. As shown in Fig. 5, depleting ETO2 (Fig. 5a) or IRF2BP2 (Fig. 5b) results in increased *Alas2*, *Epb4.2*, *Gypa* and *Slc22a4* expression levels, establishing the repressive roles of ETO2 and IRF2BP2 in regulating primed archetypical erythroid genes. This result also corroborates that ETO2 and IRF2BP2 form a functional erythroid co-repressor complex. In marked contrast, when performing the same experiments for LSD1 (encoded by the *Kdm1a* gene, Fig. 5c), which co-occupies the same genes (Supplementary Fig. 5a), either very minor changes (*Alas2*, *Gypa*, *Slc22a4*) or decreased expression (*Epb4.2*) is observed. This result, together with the data derived from the reporter assays (Fig. 2d) suggest that LSD1 does not mediate transcriptional repression by ETO2 and might even play an opposite role. To more comprehensively identify genes controlled by ETO2 and IRF2BP2, transcriptome analyses were carried out by RNA-Seq after ETO2 and IRF2BP2 depletion in MEL cells. Differentially expressed genes were also compared with the ones obtained after LSD1 depletion. Strikingly, we observe a high degree of correlation when comparing genes significantly misregulated after ETO2 or IRF2BP2 KD (Fig. 5d,e), showing that genes controlled by ETO2 are also regulated by IRF2BP2, both in a positive and negative manner. Conversely, comparison of genes misregulated in both the *Cbfa2t3* (ETO2) and *Kdm1a* (LSD1) KD shows an inverse trend, as genes repressed by ETO2 are activated by LSD1 and vice versa (Fig. 5d,f). In addition, the KD of another ETO2-interacting repressor *Gfi1b* (encoding GFI1B), which is known to interact with both ETO2 and LSD1, results in a very similar profile of differentially expressed genes when compared with the *Cbfa2t3* and *Irf2bp2* KD results (Fig. 5d). This suggests that ETO2, IRF2BP2 and GFI1B negatively regulate a set of common genes and form a repressive complex in erythroid progenitor cells. Finally, we compared misregulated genes from the ETO2-, IRF2BP2- and LSD1-depletion experiments to the gene expression changes obtained after MEL cell differentiation (Fig. 5g). The emerging correlations confirm the results presented in Fig. 5a–c: genes derepressed upon ETO2/IRF2BP2 depletion are upregulated during erythroid differentiation (including many of the primed terminal erythroid differentiation genes, Fig. 5g), while the opposite trend emerges for LSD1.

We next conducted rescue experiments in which wild-type *Cbfa2t3*/*Irf2bp2* or loss-of-interaction mutant complementary DNAs (cDNAs) were transfected into ETO2/IRF2BP2-depleted MEL cells (Fig. 5h). While the introduction of wild-type cDNA restores erythroid gene repression (Fig. 5h, orange bars), mutant cDNAs encoding ETO2/IRF2BP2 proteins no longer able to interact with each other (see Fig. 2) cannot or only partially induce transcriptional repression (Fig. 5h, grey bars). These experiments confirm that in erythroid progenitors ETO2 and IRF2BP2 function as transcriptional repressors in a highly cooperative manner. Moreover, IRF2BP2 ChIP experiments in an ETO2-deficient MEL cell line (generated using CRISPR/Cas9 technology^38^, see the Methods section) reveal a loss of chromatin binding in the absence of ETO2 (Fig. 5i). The reciprocal experiment—ETO2 ChIPs in an IRF2BP2^−/−^ MEL cell line—shows that ETO2 binding to the genome does not strictly rely on IRF2BP2 (Fig. 5i). Of note, the core LDB1 complex remains bound at the examined regulatory sites in the absence of ETO2 or IRF2BP2—ruling out the possibility of a global disruption of LDB1-complex recruitment in these cells (Supplementary Fig. 5b).

### IRF2BP2 and ETO2 repress essential erythroid pathways

**Figure 6:**
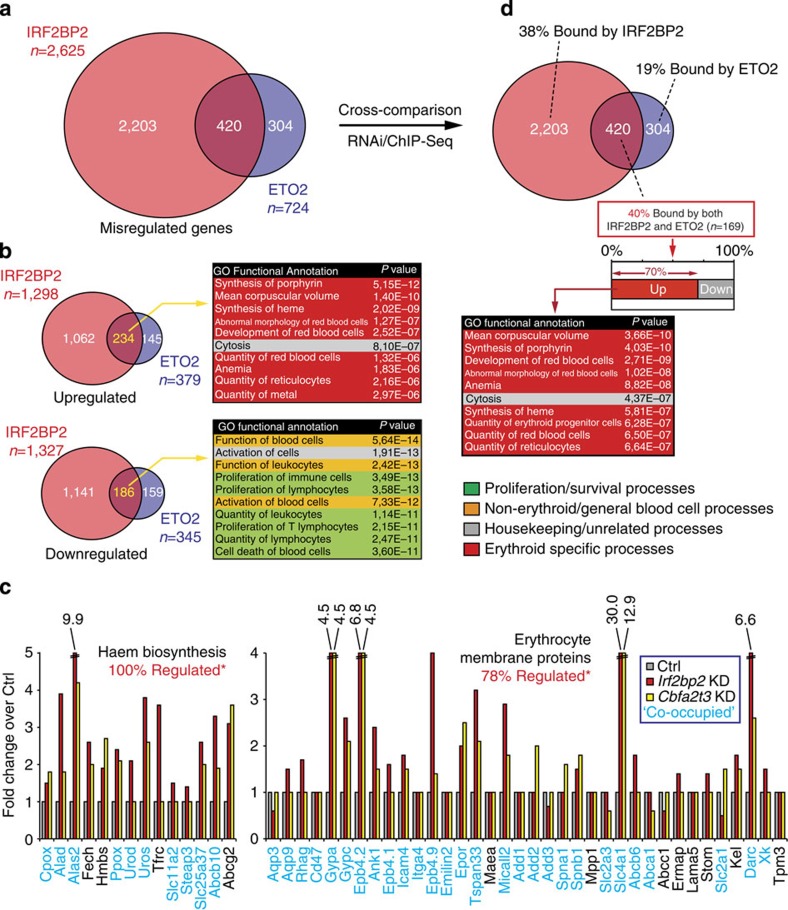
The ETO2–IRF2BP2 axis directly controls the expression of key haem biosynthesis and erythrocyte membrane proteins. (**a**) Venn diagram of differentially expressed genes (log2 FC >0.5/−0.5, *P*<0.05) after ETO2 or IRF2BP2 depletion in MEL cells. (**b**) Venn diagrams of upregulated genes (top, log2 FC >0.5, *P*<0.05) and downregulated genes (bottom, log2 FC >−0.5, *P*<0.05) after ETO2 or IRF2BP2 depletion. Genes found commonly up- or downregulated were subjected to Gene Ontology (GO) analysis using Ingenuity Pathway Analysis (IPA); the top 10 significantly associated GO functional annotations are shown. GO terms were categorized as in Fig. 3b. (**c**) Fold changes in gene expression (log2 FC >0.5/−0.5, *P*<0.05; genes not significantly affected were given a fold change of 1) of haem biosynthesis and erythrocyte membrane protein genes upon *Cbfa2t3* (encoding ETO2) and *Irf2bp2* KD. Expression levels obtained from MEL cells transduced with a non-targeting shRNA (ctrl) were set to 1. Genes bound by both ETO2 and IRF2BP2 in MEL cells (Co-occupied) are shown in blue. ‘*-% Regulated' refers to the % of total genes in the group misregulated upon *Irf2bp2* and/or *Cbfa2t3* KD. (**d**) Cross-combinatorial analysis of ETO2/IRF2BP2 ChIP-Seq data and differentially expressed genes after *Cbfa2t3*/*Irf2bp2* RNAi. Overall, 38% (IRF2BP2) and 31% (ETO2) of misregulated genes are also bound by the corresponding protein. Thirty-eight percent of the genes specifically misregulated by IRF2BP2 are bound only ‘by IRF2BP2; 19% of specific ETO2 misregulated genes are bound by only ETO2; and of the commonly misregulated genes 40% (*n*=169) was bound by both factors. Seventy percent of these co-bound/regulated genes are repressed by ETO2/IRF2BP2 and enriched for erythroid GO terms. GREAT analysis was used for assigning target genes to ChIP-Seq peaks as described in the Methods section.

To obtain functional insight into the genes affected in the *Cbfa2t3* and *Irf2bp2* KD experiments, we applied Ingenuity Pathway Analysis on the misregulated genes to link the transcriptional regulatory activities of ETO2 and IRF2BP2 to biological functions. In MEL cells, 2,625 genes are found differentially expressed upon IRF2BP2 depletion, and 724 upon ETO2 depletion. Combining these data sets, 58% of the ETO2 misregulated genes (420) are also found affected in the IRF2BP2 data set (Fig. 6a). Approximately 55% of the commonly misregulated genes are found upregulated and therefore appear to be repressed by ETO2/IRF2BP2. These 234 genes are highly enriched for erythroid functions (Fig. 6b).

In fact, 71% of the genes coding for the major components of the haem biosynthesis pathway are bound (as determined by GREAT analysis, see Methods) by ETO2 and IRF2BP2 (Fig. 6c; left graph). Furthermore, over 77% of the erythrocyte-specific membrane structural components and ion transporters are also targeted by the ETO2/IRF2BP2 complex (Fig. 6c; right graph). In correspondence with this binding pattern, almost all of the above mentioned erythroid genes are misregulated upon ETO2 and/or IRF2BP2 depletion (100% of the haem biosynthesis genes and 78% of the erythrocyte membrane proteins are affected in at least one KD, see Fig. 6c), with a strong preference for derepression. In agreement with their co-occupancy by both proteins, many genes are upregulated after either *Cbfa2t3* or *Irf2bp2* KD (71% of haem biosynthesis genes and 36% of erythrocyte membrane protein genes, see Fig. 6c). In addition, α- and β-globin gene activation was observed in both KD experiments (Irf2bp2 KD—*Hba-a1/2*: 8.2-fold up, *Hbb-b1/2*: 4.0-fold up; *Cbfa2t3* KD—*Hba-a1/2*: 3.9-fold up, *Hbb-b1/2*: 1.7-fold up). In general, 30–40% of the genes misregulated upon either *Cbfa2t3* or *Irf2bp2* KD are also bound by the corresponding factor (Fig. 6d), despite the inevitable presence of indirectly affected genes within our sets of putative targets. Strikingly, 70% of the genes bound and regulated by both ETO2 and IRF2BP2 in erythroid progenitors are found to be repressed, and these genes again exhibit a significant enrichment for erythroid functions (Fig. 6d). Together, these observations strongly indicate that the ETO2/IRF2BP2 complex controls the expression of key genes critical for erythroid cell identity and function.

ETO2 and IRF2BP2 also modulate a set of 186 genes that are downregulated upon factor depletion (Fig. 6b), of which 28% were also co-occupied. This suggests that ETO2/IRF2BP2-containing complexes can also function in gene activation. Over-represented among these are genes known to play a role in blood cell activation, proliferation and cell death (Fig. 6b). Such pathways are known to be suppressed upon erythroid differentiation and might (in part) be activated by ETO2/IRF2BP2 in progenitor cells^39^. Surprisingly, a large fraction of the over-represented processes are related to leukocyte and lymphocyte biology (Fig. 6b).

### IRF2BP2 interacts with NCOR co-repressor proteins

**Figure 7:**
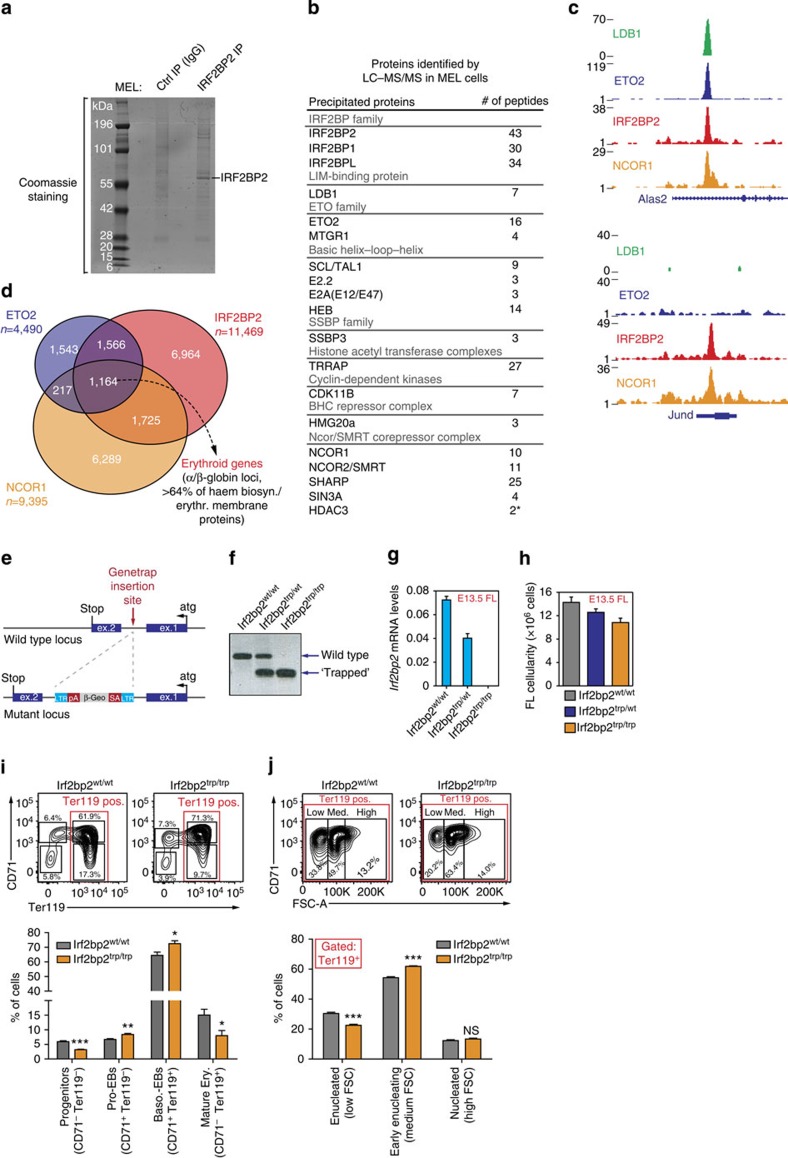
Characterization of IRF2BP2 protein partners and fetal liver erythropoiesis in IRF2BP2-deficient mice. (**a**) Coomassie staining of IRF2BP2 and control IgG-immunoprecipitated proteins separated by SDS–polyacrylamide gel electrophoresis. (**b**) IRF2BP2-interacting proteins identified by LC–MS/MS in MEL cells. Proteins pulled down in two independent experiments and with low background scores are shown, except for HDAC3 (*—only detected in one pull down). (**c**) Examples of NCOR1 recruitment to IRF2BP2-binding sites. (**d**) Venn diagram showing the genome-wide overlap between ETO2-, IRF2BP2- and NCOR1-binding sites in MEL cells. Note the significant co-localization of all three factors on the chromatin (1,164 sites), which included the α- and β-globin loci and >64% of haem biosynthesis and erythrocyte membrane protein genes shown in Fig. 6c. (**e**) A genetrap vector (containing a strong splice-acceptor (SA) and a polyadenylation sequence (pA)) was retrovirally inserted in the *Irf2bp2* intron to disrupt full-length mRNA production (genetrap allele is referred to as ‘Irf2bp2^trp^'). (**f**) Typical genotyping results obtained from a standard 3-primer PCR strategy. (**g**) *Irf2bp2* mRNA levels in whole fetal livers (FL) from E13.5 mouse embryos with the indicated genotypes (*n*=4–6 embryos per genotype, normalized to *Rnh1* levels). (**h**) Total FL cellularity in E13.5 embryos with the indicated genotypes (*n*=5–21 embryos per genotype). (**i**,**j**) Flow cytometry analysis (CD71-Ter119 double-staining) of FLs from E13.5 embryos with the indicated genotypes (*n*=9–11 embryos per genotype). Representative flow cytometry plots are shown on top; average values are plotted as bar graphs underneath. (**i**) A quadrant analysis of CD71-Ter119 staining on all live (Hoechst negative) single cells to visualize erythroid differentiation. (**j**) Ter119^+^ FL cells separated into three populations based on the FSC profile^46^. Differences between wild-type and Irf2bp2^trp/trp^ embryos were tested for statistical significance (Mann–Whitney *U*-test; *P<0.05, **P<0.01, ***P<0.001). Error bars denote s.d. NS, not significant.

Although our data strongly suggest a repressor function for IRF2BP2 in erythroid gene regulation, how IRF2BP2 achieves gene repression is still unclear. We therefore purified endogenous IRF2BP2-containing protein complexes from MEL cells and identified the interacting proteins by IP-MS. As shown in Fig. 7, we retrieve known interacting proteins such as the other IRF2BP family members^23^ and several LDB1-complex members. In addition, IRF2BP2 also interacts with proteins involved in the cell cycle and transcriptional regulation (Fig. 7b). Among the latter group are several protein complexes known to mediate transcriptional repression. Prominent among these is the nuclear receptor co-repressor/silencing mediator for retinoid and thyroid receptors (NCOR/SMRT) co-repressor complex. Key components of this complex are the NCOR1 and 2 proteins (the latter is also known as SMRT), and their repressive actions have been well documented^40^. Intriguingly, *Ncor1*^−/−^ mice die *in utero* due to abnormal erythropoiesis^41^. To test whether NCOR proteins are indeed recruited to the regulatory elements of ETO2/IRF2BP2 target genes in erythroid progenitors, we performed NCOR1 ChIP-Seq in MEL cells. This reveals a significant overlap between NCOR1- and ETO2/IRF2BP2-binding sites (1,164 sites, Fig. 7c,d). In accordance with a possible cooperative relationship between these proteins, we find that these co-occupied sites include >64% of the primed erythroid-specific genes involved in haem biosynthesis and red cell membrane function (Fig. 7c,d). Furthermore, the ETO2–IRF2BP2–NCOR1 triad occupies key regulatory elements within the α- and β-globin loci (Supplementary Fig. 5c).

We next sought to perturb NCOR1 activity to assess its contribution to ETO2/IRF2BP2 target gene repression in erythroid progenitors. Multiple attempts at depleting NCOR1 levels in MEL cells using RNA interference (RNAi) (via both lentiviral shRNA delivery and short interfering RNA (siRNA) transfections) failed to provide consistent effects on erythroid gene expression between individual shRNAs/siRNAs (*n*=3–4 for each, Supplementary Fig. 6). We believe that the numerous *Ncor1* isoforms expressed in MEL cells (as assessed by RNA-Seq, Supplementary Fig. 6) could contribute to the inconsistent effects seen with the different RNAi constructs, as alternative splicing has been reported to generate many functionally distinct NCOR1 isoforms^40^,^42^. In an attempt to inhibit NCOR1 protein function in a different manner, we decided to target the histone deacetylase 3 (HDAC3) that mediates the repressive actions of NCOR1 (reviewed in ref. 40). Apicidin is a small-molecule HDAC inhibitor reported to have specificity for HDAC3 (ref. 43), and we therefore treated MEL cells with 100 nM apicidin and measured erythroid gene expression after 24 h. Robust derepression of ETO2/IRF2BP2 target gene expression is observed after treatment (Supplementary Fig. 6), strengthening the hypothesis that NCOR1, through HDAC3, represses ETO2/IRF2BP2 target gene expression in erythroid progenitors. In addition, we observe a positive change in the ratio of LDB1/NCOR1 binding to the regulatory sequences of primed erythroid genes in differentiating MEL cells (Supplementary Fig. 6). This indicates that terminal erythroid differentiation is associated with a decreasing relative abundance of repressors such as NCOR1 as compared with activators (for example, LDB1) bound at late erythroid genes, as previously observed for ETO2 (ref. 5). Finally, an examination of published ChIP-Seq data^20^ shows that histone acetylation levels on known NCOR1/HDAC3 target residues^44^,^45^ at ETO2/IRF2BP2/NCOR1-bound erythroid genes frequently increases during erythroid differentiation (Supplementary Fig. 6), concomitant with diminished ETO2 (refs 3, 4) and IRF2BP2 (Fig. 4) expression. Together, these data indeed suggest that IRF2BP2-mediated gene repression involves the NCOR1 co-repressor complex.

### IRF2BP2-deficient mice show abnormal FL erythropoiesis

Next, we interrogated IRF2BP2 function *in vivo*. For this purpose, we used an IRF2BP2-deficient mouse model generated by a genetrap strategy (Fig. 7e). The genetrap vector (containing a strong splice acceptor) was retrovirally inserted in the *Irf2bp2* intron and results in a complete disruption of full-length messenger RNA (mRNA) production (Fig. 7e–g). Animals homozygous for the *Irf2bp2* genetrap allele (hereafter referred to as *Irf2bp2*^trp/trp^ mice) are rarely obtained and did not survive past 4 weeks of age, displaying severe growth retardation. In fact, although *Irf2bp2*^trp/trp^ embryos appear to develop normally up to E18.5 and are obtained at the expected Mendelian ratio, live births are very rare (<5% of the expected number). This indicates that *Irf2bp2*^trp/trp^ mice die either late during gestation or immediately after birth. Given that *Irf2bp2* expression is particularly high in the developing mouse lungs, skeletal muscle and heart at E17.5 (ref. 27), defects in these tissues might underlie the observed lethality. To determine whether definitive erythropoiesis is affected in these mice, we collected E13.5 FL tissue from litters obtained after crossing *Irf2bp2*^trp/wt^ mice. At this stage of murine embryonic development, the FL is the main site of definitive haematopoiesis and consists mainly of developing erythrocytes^1^. *Irf2bp2*^trp/trp^ FLs show reduced total cellularity (Fig. 7h). When stained with antibodies against the developmental CD71 and Ter119 surface markers, erythropoiesis in *Irf2bp2*^trp/trp^ FLs shows several defects (Fig. 7i). We observe a marked reduction in the double-negative immature progenitor compartment, while cells belonging to the more mature erythroblast stages (the CD71^+^ Ter119^−^ and double-positive stages) are more abundant in *Irf2bp2*^trp/trp^ FLs. Furthermore, the relative number of mature Ter119^+^CD71^−^ erythrocytes is significantly reduced in the absence of IRF2BP2. These data indicate that IRF2BP2 is important for effective FL erythropoiesis, as the output of mature erythrocytes is impaired in the absence of a functional *Irf2bp2* allele.

We further characterized terminal differentiation in *Irf2bp2*^trp/trp^ FLs by separating the Ter119^+^ population based on its forward scatter (FSC) profile^46^ (Fig. 7j). As erythroid differentiation is paralleled by a reduction in cell size, this analysis visualizes a terminal differentiation gradient ranging from large and nucleated cells (high FSC) to small, enucleated cells (low FSC). Early enucleating cells (medium FSC) are more abundantly present in IRF2BP2-deficient FLs, while the percentage of small and enucleated erythrocytes is reduced (Fig. 7j).

FL erythropoiesis at earlier developmental time points (E11.5 and E12.5) also shows altered proportions of the different developing erythroid populations in *Irf2bp2*^trp/trp^ embryos—in particular, within the mature Ter119^+^ compartment (Supplementary Fig. 7). Defects observed at E12.5 are similar in nature to the E13.5 phenotype, while at E11.5 erythropoiesis seems less severely affected: *Irf2bp2*^trp/trp^ FLs exhibit a modest decrease in pro-erythroblast abundance and an increased presence of small terminally differentiated red cells. Moreover, gene expression analysis of selected ETO2/IRF2BP2 target genes (as identified in MEL cells) in sorted FL erythroid populations reveals an upregulation of these late erythroid markers in *Irf2bp2*^trp/trp^ red cell precursors, as well as a downregulation of *Myb* expression—a marker for early erythroid progenitors (Supplementary Figs 8 and 9). Derepression of primed late erythroid genes is most striking at E11.5 and diminished at E13.5. Intriguingly, we observe a significant upregulation of the related *Irf2bpl* gene in *Irf2bp2*^trp/trp^ early erythroid precursors (Supplementary Figs 8 and 9). This phenomenon was most prominent at E13.5 and could indicate the existence of a compensatory mechanism involving IRF2BPL.

Combined, these observations point at disturbed erythroid differentiation kinetics and transcriptional regulation in the absence of IRF2BP2, confirming the notion that IRF2BP2 is important for effective erythropoiesis *in vivo*.

## Discussion

Developmental processes are coordinated by spatio-temporal changes in gene expression laid down by the combinatorial actions of TFs and the cofactors they recruit. Exactly how TFs in large multimeric complexes cooperate to create a regulatory environment that allows for rapid modulation of gene expression programmes is under intense investigation. Here we address the observation of a master haematopoietic TF complex, containing key factors required for the activation of a tissue-specific gene expression programme, that binds its target genes but maintains them in a developmental stage-specific primed state. Previous studies have shown that the activating LDB1 TF complex is already recruited to genes of the late erythroid-specific transcriptome in erythroid progenitors, before their full activation^3^,^4^,^5^,^6^,^7^. We have further confirmed and characterized the transcriptional and epigenetic status of these primed erythroid genes, revealing that they reside in active chromatin and are not controlled through an RNAPII pause–release mechanism^19^. One particular complex member, the ETO2 co-repressor, was found to mediate priming by repressing LDB1-complex target gene expression^3^,^4^,^5^,^7^. ETO2-mediated repression remains poorly understood, although the GFI1B TF, HDACs and the SIN3 transcription regulator family member A (Sin3A) repressor protein have been implicated (either directly or via their interaction with TAL1)^3^,^30^,^47^. We set out to further investigate the molecular mechanisms used by ETO2 to suppress terminal erythroid gene expression in progenitor cells.

A proteomics approach was first used to catalogue ETO2-interacting proteins in MEL erythroid progenitors (Fig. 1), identifying several repressor candidates known to bind ETO2 or other LDB1-complex members (for example, GFI1B^3^ and LSD1 (ref. 22)). Interestingly, we also detect the IRF2BP2 co-repressor in our interaction screen. Follow-up experiments firmly establish a cooperative role for ETO2 and IRF2BP2 in maintaining the late erythroid transcriptional programme in a primed state in undifferentiated progenitors: (1) IRF2BP2 strongly enhances ETO2-mediated repression *in vitro*, which is fully dependent on the ETO2–IRF2BP2 interaction (Fig. 2); (2) ETO2 and IRF2BP2 chromatin occupancy shows extensive genome-wide co-localization at genes involved in red blood cell development and function (Fig. 3); (3) similar to *Cbfa2t3* (ETO2), *Irf2bp2* expression is reduced upon erythroid differentiation, concomitant with the upregulation of its erythroid target genes (Fig. 4); (4) depletion of ETO2 or IRF2BP2 leads to overlapping effects on gene expression, in particular the strong derepression of the late erythroid-specific transcriptome (Fig. 5); (5) ETO2/IRF2BP2 mutant proteins unable to interact with each other show impaired induction of erythroid gene repression (Fig. 5); (6) ETO2 and IRF2BP2 bind the regulatory regions of >70% of the critical haem biosynthesis and erythrocyte membrane genes, the majority of which are repressed by both factors (Fig. 6).

Our biochemical analyses of IRF2BP2 protein complexes in MEL cells reveal the presence of NCOR/SMRT co-repressor complex members (Fig. 7b). In accordance, a key component of this complex, NCOR1, shows extensive genomic co-occupancy with IRF2BP2 and the ETO2/LDB1 complex in erythroid progenitors (Fig. 7d). Among these co-occupied sites, we find the vast majority of ETO2/IRF2BP2-repressed erythroid genes. Inhibition of NCOR1 complex activity by the apicidin inhibitor results in the upregulation of genes repressed by ETO2 and IRF2BP2 (Supplementary Fig. 6). On the basis of these data, we propose that IRF2BP2 confers repression upon ETO2/LDB1-complex target genes in part via its interaction with the NCOR/SMRT co-repressor complex. In accordance with our hypothesis, NCOR1-deficient mice showed abnormal FL erythropoiesis and developed severe anaemia during mid-gestation^41^.

We have also investigated the role of other ETO2-interacting putative repressor proteins. Although we could not detect Sin3A in our ETO2 IPs from erythroid cell lines, we did find the GFI1B TF and the LSD1 lysine demethylase, both of which have been implicated in the repression of LDB1-complex target genes^3^,^22^,^48^. Both proteins colocalize with the ETO2-containing LDB1 complex on the erythroid progenitor genome (Supplementary Fig. 5). In discordance with the findings of Hu *et al*.^22^, we find no evidence for LSD1-mediated repression of the erythroid-specific *Epb4.2* gene (Fig. 5). In fact, we observe the opposite effect of LSD1 depletion on the late red cell transcriptome when compared with the *Cbfa2t3*/*Irf2bp2* KDs (Fig. 5), similar to the loss of erythroid marker expression and differentiation upon LSD1 KD reported by Saleque *et al*.^49^. We conclude that LSD1, as part of the LDB1 complex, in general fulfills an activating role in erythroid differentiation (that is, possibly through controlling H3K4 methylation status^50^). In contrast, GFI1B, a DNA-binding repressor previously found to be required for terminal erythroid differentiation^51^, appears to repress LDB1-complex target genes in a similar manner as ETO2/IRF2BP2 (Fig. 5d). As was reported for ETO2, interactions between GFI1B and the activating LDB1-complex member TAL1 were strongly diminished upon terminal erythroid differentiation^3^. Cooperation of GFI1B with ETO2 and IRF2BP2 seems a plausible scenario warranting further investigation.

Intriguingly, IRF2BP2 binds many genomic regions independent of ETO2 and the LDB1 complex (Fig. 3). IRF2BP2-only sites are generally located closer to TSSs and therefore appear more frequently involved in short-range or promoter-based gene regulation as compared with LDB1-complex-associated IRF2BP2 sites (Supplementary Fig. 4). Furthermore, IRF2BP2 depletion affected the expression of numerous genes in an ETO2-independent fashion (Fig. 6). These observations suggest that IRF2BP2 plays additional roles in erythroid progenitors, independent of ETO2 and the LDB1 complex. In such cases, targeting of IRF2BP2 to the DNA could be mediated by ETS, TCF and EGR/SP family TFs, as binding motifs for these factors are strongly enriched at sites only bound by IRF2BP2 (Fig. 3c,d). Surprisingly, we did not detect a significant enrichment of IRF-binding motifs at these regions, nor did we find IRF TFs interacting with IRF2BP2 in our MS experiments. IRF2BP2 was originally identified as an IRF2-interacting factor in a yeast two-hybrid screen^23^. An IRF2–IRF2BP2 complex was recently detected in the K562 human erythroleukaemia cell line^52^, and IRF2 is expressed in MEL and primary murine erythroid progenitor cells (>5.0 RPKM as measured by RNA-Seq). Whether this discrepancy reflects a species–specific difference or differences in experimental systems is unclear. Nevertheless, our combined analysis of IRF2BP2-binding sites and protein partners does provide preliminary insight into the ETO2/LDB1-independent functions of IRF2BP2. Genes bound only by IRF2BP2 are significantly enriched for functions related to proliferation and apoptosis (Fig. 3b), and the cell-cycle regulator cyclin-dependent kinase 11B interacts with IRF2BP2 (Fig. 7b). Interestingly, several studies have implicated IRF2BP2 in the regulation of cell survival^25^,^26^,^53^.

In agreement with our experiments in MEL cells, IRF2BP2 also appears to be important for erythropoiesis and erythroid gene regulation *in vivo*. Perinatal lethality of IRF2BP2-deficient mice precluded the analysis of adult erythropoiesis in our *Irf2bp2* genetrap model. However, analysis of mid-gestation-definitive FL erythropoiesis in these mice shows that IRF2BP2 is required for an effective output of terminal erythroid differentiation (Fig. 7i,j; Supplementary Fig. 7b). The exact nature of this defect remains to be determined, but our experiments indicate the presence of a partial differentiation block at the erythroblast stage, before enucleation (Fig. 7i,j). Alternatively, the observed erythroblast expansion could be a consequence of accelerated progenitor differentiation or represent a compensatory mechanism, which could also explain the partially exhausted progenitor compartment observed at E13.5 (Fig. 7i) and the premature silencing of the immature *Myb* marker^54^ (Supplementary Figs 8 and 9). Gene expression analyses on purified erythroid progenitor populations from wild-type and *Irf2bp2*^*trp/trp*^ embryos reveal a premature upregulation of late erythroid markers in IRF2BP2-deficient progenitors that is significantly more pronounced at E11.5 as compared with E13.5 (Supplementary Figs 8 and 9). Intriguingly, we observe a strong upregulation of *Irf2bpl* in *Irf2bp2*^*trp/trp*^ progenitors and a delayed extinction of *Irf2bpl* expression during *in vivo* erythroid maturation—in particular at E13.5 (Supplementary Figs 8 and 9). As IRF2BP2 and IRF2BPL reside in the same protein complexes (Fig. 7b), we hypothesize that elevated IRF2BPL levels might compensate for a loss of IRF2BP2 *in vivo*. Future investigations will reveal whether the other IRF2BP family members indeed play a role in erythropoiesis and in the regulation of primed erythroid gene expression. While this paper was under review, IRF2BP2 was shown to be important for macrophage-mediated inflammation^55^, suggesting that IRF2BP2 may have a multifaceted role in blood cell development and function.

In summary, we show that the control of developmentally primed erythroid genes depends on the cooperative actions of ETO2 and its novel binding partner IRF2BP2. Repression by the ETO2–IRF2BP2 axis is lost during erythroid differentiation, resulting in the full activation of the late erythroid-specific transcriptome by the LDB1 complex. These results provide new insight into the control of lineage-specific transcriptional programmes, as they suggest that an intricate balance between the activating and repressive components of a TF complex underlies the implementation of lineage-specific gene expression. Furthermore, using an IRF2BP2-deficient mouse model, we confirm the relevance of a functional *Irf2bp2* allele for effective erythropoiesis *in vivo*.

## Methods

### Cell culture and Irf2bp2 genetrap animals

MEL (C88 clone^56^) and HEK 293T (ATCC) cells were maintained in DMEM containing 10% FCS and penicillin/streptomycin. ETO2-V5-Bio MEL cells expressing BirA were generated and maintained as described previously^5^,^21^. Apicidin (Calbiochem, 178276) was reconstituted in dimethylsulphoxide to obtain a 10mM stock. MEL cells were treated by adding Apicidin directly to the medium to a final concentration of 100 nM. *Irf2bp2*^trp/wt^ C57BL/6 ES cells were produced by the Texas A&M Institute for Genomic Medicine (College Station, TX) through the insertion of a genetrap construct in the first intron of the *Irf2bp2* gene (clone IST11591C1). Genetrap location was verified using standard PCR and sequencing methods. Mouse ES cells were injected into blastocysts and implanted into pseudopregnant albino fosters according to standard methods. Chimeric animals were further crossed to obtain heterozygous founders and mice were further crossed on a mixed-FVB/N-C57BL/6 background. Mice were genotyped using a standard 3-primer PCR method. Up to E18.5 of embryonic development, wild-type, heterozygous and homozygous genetrap animals were obtained at the expected Mendelian ratio and with no visible signs of developmental defects. Although *Irf2bp2*^trp/wt^ animals were born in normal numbers, *Irf2bp2*^trp/trp^ mice were rarely born (<5% of the expected number). Homozygous animals showed severe growth retardation and all died the first weeks after birth. Ethical approval was obtained from the Committee on the Ethics of Animal Experiments (DEC) of the Erasmus MC as well as the French Ministry of Agriculture regulations (DDPP92 animal facility registration number: B 92-032-02). All animal experiments were carried out according to institutional and national guidelines.

### (Co-)Immunoprecipitations and MS analysis in MEL cells

Protocols for the preparation of nuclear extracts, streptavidin-mediated protein capture and LC–MS/MS in MEL cells have been described previously^21^. On-bead proteolytic digestion was performed by rapidly washing the beads containing bound ETO2-V5-Bio complexes twice with trypsin digestion buffer (50 mM NH_4_HCO_3_), after which to each 50 μl of beads 100 μl of trypsin digestion buffer and 10 μl of trypsin solution (10 μg ml^−1^) were added. Samples were incubated overnight at 37 °C with shaking. Digested samples were analysed by nanoflow LC–MS/MS on a LTQ-Orbitrap (Thermo) mass spectrometer coupled to an 1100 series LC pump and autosampler (Agilent), operating in positive mode and equipped with a nanospray source. Peptide mixtures were trapped on a ReproSil C18 reversed-phase column (Dr Maisch GmbH; column dimensions 1.5 cm × 100 μm, packed in-house) with a flow rate of 8 μl min^−1^. Peptide separation was performed using a ReproSil C18 reversed-phase column (Dr Maisch GmbH; column dimensions 15 cm × 50 μm, packed in-house) using a linear gradient from 0 to 80% B (*A*=0.1 M formic acid; *B*=80% (v/v) acetonitrile, 0.1 M formic acid) over 70 min with a constant flow rate of 200 nl min^−1^ using a splitter. The column eluent was directly sprayed into the electrospray ionization source of the mass spectrometer. Mass spectra were acquired in continuum mode; while fragmentation of the peptides was performed in a data-dependent mode. Peak lists were automatically created from raw data files using the Mascot Distiller software (version 2.1; MatrixScience). The Mascot search algorithm (version 2.2, MatrixScience) was used for searching against the NCBInr database (latest NCBInr release; taxonomy: *Mus musculus*). The peptide tolerance was typically set to 10 p.p.m. and the fragment ion tolerance to 0.8 Da. A maximum number of two missed cleavages by trypsin were allowed and carbamidomethylated cysteine and oxidized methionine were set as fixed and variable modifications, respectively. The Mascot score cutoff value for a positive protein hit was set to 60. Individual peptide MS/MS spectra with Mowse scores below 40 were checked manually and either interpreted as valid identifications or discarded. Identified proteins were filtered against a database of background hits obtained from BirA-expressing MEL cells. Proteins with no peptides identified in the BirA control experiment or showing Mascot scores at least threefold higher than the control were considered as positive hits, and were further validated by conventional co-IP experiments (Fig. 1d; Supplementary Fig. 2b) For endogenous co-IPs, 0.5 mg of MEL nuclear extracts were diluted to reach 100 mM KCl salt concentration using Heng 0 buffer (20 mM HEPES KOH pH 7.9, 20% glycerol, 0.25 mM EDTA, 0.05% NP-40). Extracts were treated with 1 U Benzonase nuclease (Millipore). Protein extracts were incubated with the specific antibody overnight at 4 °C, followed by addition of protein A or G Sepharose bead slurry (50 μl slurry per IP; Millipore) and incubation at 4 °C for 1 h. Beads were pelleted, washed three times in Heng 100 buffer (Heng buffer containing 100 mM KCl) and boiled for 5 min at 95 °C in Laemmli buffer before being subjected to western blot analysis. Proteomics analysis of endogenous ETO2- and IRF2BP2-interacting proteins was carried out by direct immune capture as described previously^57^. Briefly, purification of endogenous protein complexes was performed by crosslinking 10 μg antibody (see below), or control immunoglobulin to 50 μl protein G Sepharose beads (Amersham). Antibody–bead complexes were blocked with 0.1 mg ml^−1^ insulin (Sigma), 0.2 mg ml^−1^ chicken egg albumin (Sigma) and 1% fish skin gelatin (Sigma) for 1 h at room temperature and directly added to 1.5 ml of MEL nuclear extracts containing benzonase. After 3 h incubation at 4 °C, antibody–bead complexes were washed five times in C-100 buffer (20 mM Hepes pH 7.6, 20% glycerol, 100 mM KCl, 1.5 mM MgCl_2_, 0.2 mM EDTA, 0.02% NP-40) and boiled in Laemmli buffer. Proteins were loaded on a 4–12% acrylamide gel and lanes were cut for LC–MS/MS analysis as described above. For MS analysis of ETO2- and IRF2BP2-interacting proteins, two independent biological replicates (for both experimental and control samples) were analysed to ensure reproducible and specific binding partner identification. The following antibodies were used: ETO2 G-20 (Santa Cruz, sc9741), an IRF2BP2 rat monoclonal KT139 (clone 10G3, produced by Absea Antibodies, Beijing), GFI1B B-7 (Santa Cruz, sc8559), LDB1 N-18 (Santa Cruz, sc-11198), LSD1 (Abcam, ab17721), RUNX1 H-65 (Santa Cruz, sc28679), E2A V-18 (sc-349), HEB A-20 (sc-357), SSBP3 (Abcam, ab83815), V5 (Invitrogen, R960-25), Flag M2 (Sigma, F1804) and haemagglutinin (HA) (Sigma, H6908).

### Co-IPs and luciferase assays in HEK 293T cells

HEK 293T cells were transfected with Lipofectamine 2000 (Invitrogen) according to the manufacturer's instructions. For ETO2–IRF2BP2 interaction domain mapping, we constructed a series of Flag-tagged ETO2-deletion mutants, V5-IRF2BP2 and the HA-IRF2BP2deltaRING-deletion mutant (see Fig. 2) in the pcDNA3.1 expression vector (Invitrogen). HEK 293T cells were lysed 48 h post transfection in whole-cell lysis buffer (20 mM HEPES KOH pH 7.5, 150 mM KCl, 10% glycerol, 2.5 mM EDTA, 5 mM dithiothreitol, 0.1% Triton X-100 (Sigma) and protease inhibitor cocktail (Roche)). Extracts were treated with 1 U Benzonase nuclease (Millipore). Protein extracts were incubated with the anti-Flag, anti-V5 or anti-HA antibodies overnight at 4 °C, followed by addition of protein A or G Sepharose bead slurry (50 μl slurry per IP; Millipore) and incubation at 4 °C for 1 h. Beads were pelleted, washed three times in lysis buffer and boiled for 5 min at 95 °C in Laemmli buffer before being subjected to western blot analysis. Full-length *Kdm1a* (LSD1) cDNA was cloned in pcDNA3.1 for reporter assay experiments. The Gal4–ETO2 fusion protein was generated by fusing full-length *Cbfa2t3* cDNA sequence to a Gal4 DNA-binding domain in pcDNA3.1. The Gal4-responsive firefly luciferase plasmid was a kind gift from Dr Jan van der Knaap (Erasmus MC). A Renilla luciferase expressing vector (pRL-TK, Promega) was co-transfected and used for normalization. Luciferase assays were performed using the Dual-Luciferase Reporter Assay System (Promega) according to the manufacturer's instructions^54^. For rescue experiments, MEL cells transduced with shRNAs against *cbfa2t3* (ETO2) or *Irf2bp2* were transfected with expression vectors containing shRNA-immune cDNAs encoding ETO2, IRF2BP2 or mutant versions of these proteins using GeneCellin (BioCellChallenge). RNA extractions and gene expression analysis by qPCR were carried out 48 h after transfection.

### ChIP and ChIP-Seq experiments

Protocols for the preparation of chromatin from MEL cells, IP and sample preparation for Illumina sequencing have been previously described in great detail^5^,^21^. For NCOR1 ChIP-Seq, 10^8^ MEL cells were crosslinked with 2 mM disuccinimidyl glutarate (Thermo Fisher Scientific) and 1% formaldehyde as previously described^58^. Antibodies used for ChIP (10 μg per experiment) are identical to those used for IP (detailed above), except for GFI1B (D-19 Santa Cruz, sc8559). Reads were mapped against NCBI build 37.1 of the mouse genome (mm9) using Bowtie (version 2.0.0)^59^. Uniquely mapped reads were extended to 200 bp in the 3′ direction and were transformed into a genome-wide read density (coverage) using custom R scripts. MACS (version 1.4.2)^60^, CCAT (version 3.0)^61^ and in-house peak-calling software (available from the GitHub repository, https://github.com/supatt/rChIPSeqTools.git) with default parameters were used to comprehensively identify binding sites. We combined binding sites identified by all three methods to define consensus-binding regions using GenomicRanges. Binding regions predicted by each program were profiled on each genomic location. We assigned value ‘1' to the genomic locations that overlapped with the predicted binding sites, and we assigned ‘0' to the non-overlapping locations. We then generated the whole-genome coverage vectors from all binding regions of all methods and summed up these coverage vectors. We selected only genomic regions that had a summed up coverage value ⩾2 and a minimum region length ⩾150 bp as candidate consensus-binding regions. Consensus-binding regions were given *P* values based on a negative binomial distribution as modified from PeakSeq^62^ and assigned *P* values were adjusted using the Benjamini–Hochberg method. Candidate binding sites were then selected for the downstream analysis based on the following criteria: read counts ⩾10 reads, fold changes (FCs) ⩾2 compared with immunoglobulin (Ig)G control and adjusted *P* values ≤0.01. To classify co-binding patterns, ETO2- and IRF2BP2-binding sites were combined using GenomicRanges (using the ‘findOverlaps' function with the minimum overlap regions=250 bp from the peak centre). Binding signal coverage for each site was then normalized to obtain equal levels of background signal in both antibody and IgG control experiments (normalization method was modified from Peakseq^62^). Normalized coverage for the IgG control experiment was subtracted from the normalized coverage for the Antibody experiment. We next retrieved the subtracted coverage within±0.5 kb relative to the centre of each binding site and calculated the standard *z*-scores in each sub-window (25 bp). The matrix of standard *z*-scores per individual binding site was then subjected to *K*-means (*K*=3) clustering. Clustering analysis results were visualized with Java Treeview^63^. After *K*-means clustering, we selected representative binding sites for each co-binding pattern (1,760 for ETO2-only, 2,730 for ETO2/IRF2BP2 and 5 random sets of 4,000 IRF2BP2-only). We retrieved repeat-masked 200-bp DNA sequences centred on each binding site and removed the binding sites that contain ⩾150 bp repeat-masked DNA. We next performed *de novo* motif discovery using MEME^64^ with a minimum motif width of 5 bp and a maximum number of 10 motifs as the required parameters. We excluded motifs supported by ≤100 sites and removed repeat motifs. ‘IRF2BP2-only' category motifs were selected based on their consistent identification in the five random sets. Results from MEME were subjected to an in-house ChIP-Seq analysis pipeline (available from the GitHub repository, https://github.com/supatt/rChIPSeqTools.git) to generate motif logos and to calculate the proportion of motif containing sites. Derived motifs were then compared with known motifs in the JASPAR database^65^ using Tomtom^66^ with parameters: ‘-min-overlap 4 -dist pearson -evalue -thresh 0.05'. Online GREAT^31^ was used to assign TF-binding sites to genes and identify associated biological processes. Different GREAT analysis parameters were tested and yielded highly comparable results. Results using the ‘single nearest gene method (within 1 Mb)' parameter are shown.

### Real-time qPCR

For gene expression analysis, RNA extractions were performed using TRIPure (Sigma) and cDNA synthesized using SuperScript II reverse transcriptase and Oligo(dT) primers (Invitrogen). ChIP DNA or cDNA were used as template in triplicate qPCR reactions (Platinum Taq DNA polymerase, Invitrogen) and analysed on a CFX96 system (Bio-Rad). SYBR Green (Invitrogen) was used for quantification. Gene expression values were normalized to *Rnh1* mRNA levels^54^. Primer sequences can be found in Supplementary Table 1.

### RNAi and CRISPR/Cas9-engineered deletions

Lentivirus particles were produced as described^54^. shRNA sequences were obtained from the MISSION TRC shRNA library (Sigma, for *Kdm1a* and *Ncor1*), designed manually and cloned into pLKO.1 (for *Irf2bp2*; sh1: 5′- CTCCAGACAAAGCATTAAA -3′ and sh3: 5′- CAACGGGTCTAAAGCAGTT -3′) or described before (*Cbfa2t3* (ref. 5)). For *Gfi1b* KDs, MEL cells were transfected with FlexiTube *Gfi1b* siRNA #1 and #7 (SI01011227 and SI05169871, Qiagen) using HiPerfect transfection reagent (Qiagen) according to the manufacturer's instructions. Non-targeting shRNAs/siRNAs were used as controls. Cells were harvested 48 or 72 h after transduction/transfection and processed for RNA/protein extraction as described above. The generation of knockout cell lines by CRISPR/Cas9 technology was carried out as previously described^67^ using the pSpCas9(BB)-2A-Puro (PX459) vector (obtained from Addgene). Small guide RNA sequences for targeted inactivation of *Cbfa2t3* and *Irf2bp2* in MEL cells were designed using the CRISPOR webtool (http://crispor.tefor.net/crispor.cgi). Combinations of two different guide RNAs were used for each gene to delete critical exons (*Cbfa2t3*: 5′- GACTGGGGCCTCACAAACGA -3′ and 5′- GAACGGTTGCAGGGACAGAG -3′; *Irf2bp2*: 5′- GGTCAACGGTTCTGCCGCGC -3′ and 5′- GGCTTTCCTGCTGACCAGCC -3′). MEL cells were transfected using Genecellin (BioCellChallenge), single clones were isolated and targeted genomic deletions were verified by PCR and sequencing.

### RNA sequencing

Total RNA was extracted from MEL or E13.5 sorted FL populations using the RNeasy mini kit (Qiagen). After qPCR validation, RNA was used for mRNA-sequencing on an Illumina HiSeq 2000 (standard TruSeq RNA-sequencing protocol). At least two independent biological replicate samples were sequenced and used for downstream analysis. Raw reads were mapped with Tophat2 (version 2.0.10)^68^ against mouse genome NCBI build 37.1 (mm9) with default parameters using ‘no-coverage-search', and ‘segment-length 18' as input options. We next quantified the expression per UCSC RefSeq (mm9) gene to obtain read count and RPKM values using the *rpkmforgenes* script. The non-adjusted read counts for each gene were used for statistical calculation of global differential expression using DESeq2 (ref. 69). Differentially expressed genes were selected at an adjusted *P* value of ≤0.05 (Benjamini–Hochberg corrected). We selected differentially expressed genes with log2 FCs ⩾0.5 and log2 FC ≤−0.5 in each KD data set to generate the correlation plots shown in Fig. 5. We selected the top 142 differentially expressed genes (log2 FC ⩾1 and log2 FC ≤−1) from the *Cbfa2t3* KD data set for the clustering analysis. We then retrieved all log2 FC values for the 142 genes in the other KD data sets. Hierarchical clustering and visualization were performed using MeV (ref. 70). For Ingenuity Pathway Analysis (Qiagen), only genes with a log2 FC ⩾0.5 or ≤−0.5 and a *P*≤0.05 were considered. Core Analysis (standard settings) was used to extract Gene Ontology terms that were associated with a gene set in a statistically significant fashion.

### Flow cytometry

E11.5–13.5 embryos were harvested and dissected to collect the FL. Hundred microlitre of PBS containing 1 million single cells obtained from whole E13.5 FLs were stained with 5 ng μl^−1^ CD71-FITC (553266) and Ter119-PE (553673) antibodies (BD Pharmingen). Hoechst was used as a viability dye (Sigma), and cells positive for Hoechst staining were excluded from further analysis (>70% of the total cell population consisted of viable cells). Flowcytometric analysis was performed using a BD LSRFortessa flowcytometer (BD Biosciences), collecting a minimum of 10,000 events per sample. FACS sorting was performed with a BD FACSAria III (BD Biosciences) using the above described staining protocol. At least 1 million cells were sorted for RNA extraction.

### Immunofluorescence

MEL cells were fixed on poly-prep glass slides (Sigma) and fixed in 4% paraformaldehyde for 15 min at room temperature. Cells were permeabilized with 0.1% Triton X-100, blocked with 0.5% bovine serum albumin/0.15% glycin (in PBS) and incubated overnight with ETO2 or V5 antibodies at 4 °C. After a 2-h incubation with appropriate secondary antibodies at room temperature, coverslips were mounted on glass slides with Vectashield (+DAPI, Vector Laboratories).

### Published genome-wide data sets used

The following publicly available data sets were used: LDB1, GATA1 and ETO2 ChIP-Seq data (MEL, SRA ERA000161 (ref. 5)); RNA-Seq data (MEL/G1E/G1E-ER, ENCODE Penn State University; available at the UCSC Genome Browser (mouse genome, mm9)); p300 ChIP-Seq data (MEL, ENCODE Stanford/Yale; available at the UCSC Genome Browser (mouse genome, mm9)); H3K27Ac ChIP-Seq data (MEL/FL E14.5/Brain, ENCODE Ludwig Institute for Cancer Research; available at the UCSC Genome Browser (mouse genome, mm9)); DNAse I-Seq data (MEL/FL E14.5/Brain, ENCODE University of Washington; available at the UCSC Genome Browser (mouse genome, mm9)); GATA1, TAL1 and RNAPII ChIP-Seq data (MEL/G1E/G1E-ER, ENCODE Penn State University; available at the UCSC Genome Browser (mouse genome, mm9)); microarray gene expression data (Fetal and adult erythroid populations, ErythronDB database online^35^).

## Additional information

**Accession codes:** Nucleotide sequences for ChIP-Seq and RNA-Seq data sets have been deposited in the Gene Expression Omnibus (GEO) database under accession code GSE59859. The mass spectrometry proteomics data have been deposited in the ProteomeXchange Consortium/PRIDE partner repository under accession code PXD001892.

**How to cite this article:** Stadhouders, R. *et al*. Control of developmentally primed erythroid genes by combinatorial corepressor actions. *Nat. Commun.* 6:8893 doi: 10.1038/ncomms9893 (2015).

## Supplementary Material

Supplementary Information Supplementary Figures 1-11, Supplementary Table 1 and Supplementary References
