# Supplementary material for: Control of developmentally primed erythroid genes by combinatorial co-repressor actions
Source: Nat Commun. 2015 Nov 23;6:8893. doi: 10.1038/ncomms9893 (PMC4673834; doi:10.1038/ncomms9893)
Supplement: Supplementary Information — Supplementary Figures 1-11, Supplementary Table 1 and Supplementary References [file ncomms9893-s1.pdf]

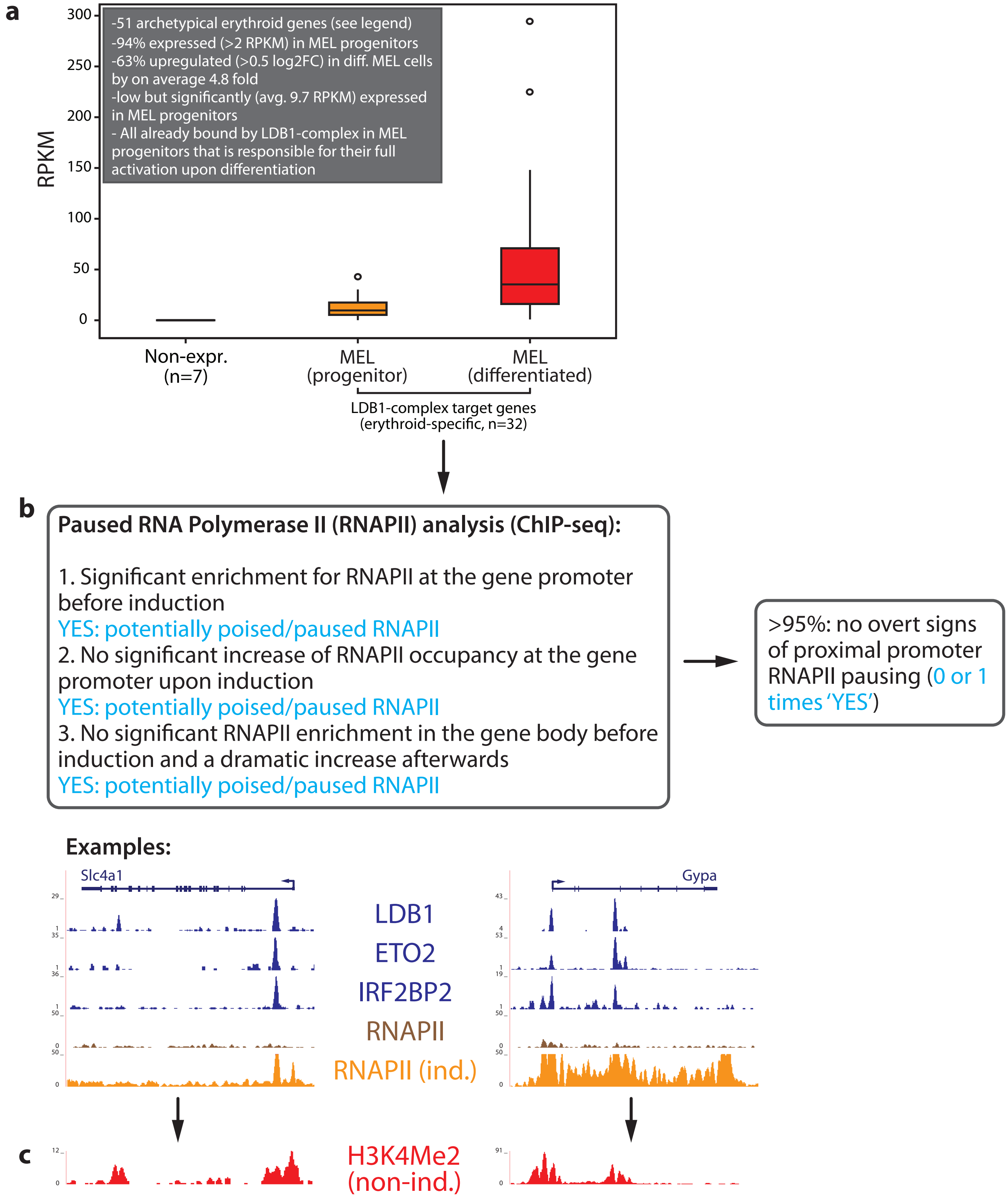


**Supplementary Figure 1. Transcriptional and epigenetic definition of primed LBD1-complex target genes.** (a) Quantification box plot depicting the average expression levels (RPKM) of non-expressed genes ('Non-expr.) and 51 archetypical erythroid genes (involved in heme biosynthesis and erythrocyte membrane function[1]) in MEL cells before ('progenitor') and after ('differentiated') induction of erythroid maturation. Various characteristics of this gene set during MEL cell differentiation are outlined in the gray box. (b) LDB1-complex target genes (n=32, as defined by ChIP-Seq) from the erythroid gene set used in panel a were inspected for RNA Polymerase II (RNAPII) enrichment (measured by ChIP-Seq) during MEL cell differentiation. Three criteria were defined that indicate the presence of paused RNAPII at the proximal promoter of these genes. Genes scoring a positive response for 2 or 3 criteria were defined as 'paused'. Examples of two of the examined archetypical erythroid genes (*Slc4a1* and *Gypa*) are shown, both showing no overt signs of RNAPII pausing. (c) H3K4Me2 ChIP-Seq profiles from non-induced MEL progenitor cells for the *Slc4a1* and *Gypa* loci shown in panel b. ind.: induced MEL, non-ind.: non-induced MEL

a

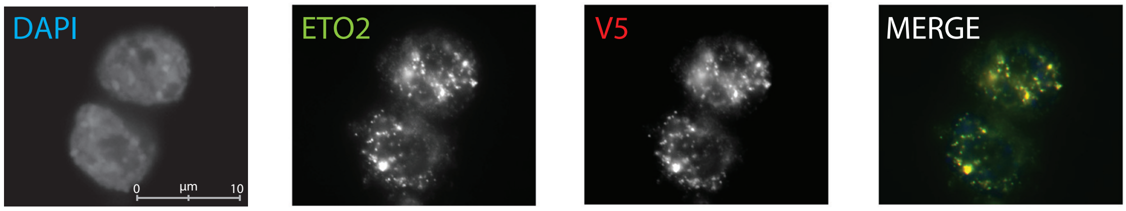


b

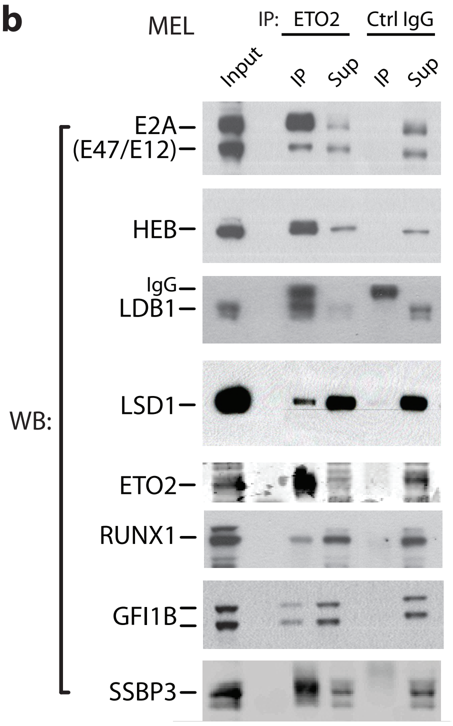


c

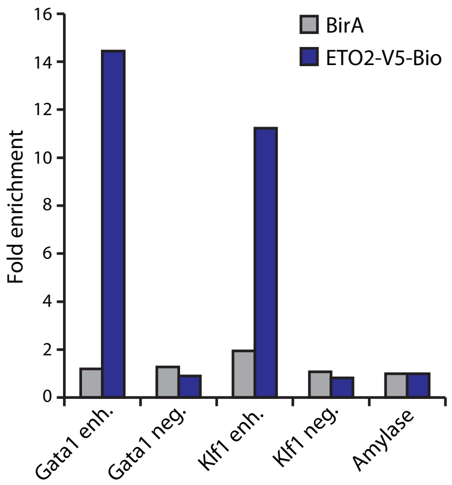


d

Proteins identified by LC-MS/MS in non-induced MEL cells

| Precipitated proteins | ETO2-V5-Bio Streptavidin pulldown | | Endogenous ETO2 pulldown | |
|---|---|---|---|---|
| | nb unique peptides ETO2-V5-Bio | nb unique peptides BirA | nb unique peptides ETO2 IP | nb unique peptides IgG IP |
| LIM binding protein | | | | |
| LDB1 | 6 | 0 | 12 | 2 |
| ETO family | | | | |
| ETO2 | 7 | 0 | 29 | 0 |
| MTGR1 | 6 | 0 | 21 | 0 |
| Basic helix-loop-helix | | | | |
| LYL1 | 4 | 0 | 1 | 0 |
| SCL/TAL1 | 7 | 0 | 15 | 3 |
| E2.2 | 4 | 0 | 14 | 0 |
| E2A (E12/E47) | 13 | 0 | 21 | 0 |
| HEB | 19 | 0 | 19 | 0 |
| SSBP family | | | | |
| SSBP2 | 5 | 0 | 5 | 0 |
| SSBP3 | 4 | 0 | 5 | 0 |
| SSBP4 | 3 | 0 | 3 | 0 |
| Transcriptional activator | | | | |
| FUS/TLS | 2 | 0 | nd | |
| Repressor / co-repressor | | | | |
| IRF2BP2 | 6 | 0 | 4 | 0 |
| GFI1B | 2 | 0 | nd | |
| Runt related | | | | |
| Runx1 | 4 | 0 | 8 | 4 |
| CBF-beta | 1 | 0 | 2 | 0 |
| BHC repressor complex | | | | |
| LSD1 | 13 | 2 | 10 | 3 |
| GSE1 | 9 | 1 | 5 | 1 |
| REST co-repressor 3 | 4 | 0 | nd | |
| HMG20a | 2 | 0 | nd | |
| HDAC2 | 3 | 0 | nd | |
| Jumonji-domain demethylase 1C/2C | 1 | 0 | nd | |

**Supplementary Figure 2. Validation of ETO2-V5-Bio functionality in MEL cells.** (a) MEL cells stably expressing BirA and ETO2-V5-Bio cells were fixed and stained for endogenous ETO2 (in green) or for ETO2-V5-Bio (using a V5 antibody, in red). Note the nuclear localization (as compared to the DAPI nuclear stain) of ETO2-V5-Bio and co-localization with endogenous ETO2. (b) Endogenous co-immunoprecipitation validations of ETO2-V5-Bio interacting proteins in MEL cells identified by LC-MS/MS. Species-matched IgG was used to control for non-specific binding. Full-size blot images can be found in Supplementary Fig.10. (c) Bio-ChIP qPCR experiments showing recruitment of ETO2-V5-Bio to known endogenous ETO2 genomic binding sites (*Gata1* -3.5 HS enhancer and the *Klf1* upstream enhancer). Regions immediately up- or downstream (+ or – 1kb) of the enhancer (enh.) sites were used as negative (neg.) controls. Enrichments were normalized to Amylase promoter values. A representative of two independent experiments is shown. (d) A comparison between proteins identified by mass spectrometry using the ETO2-V5-Bio streptavidin pulldown and the endogenous ETO2 pulldown. See Methods section for further details on methodology and analysis. Full-size images of all Western Blots shown can be found in Supplementary Fig.10. WB, Western Blot; IP, immunoprecipitation

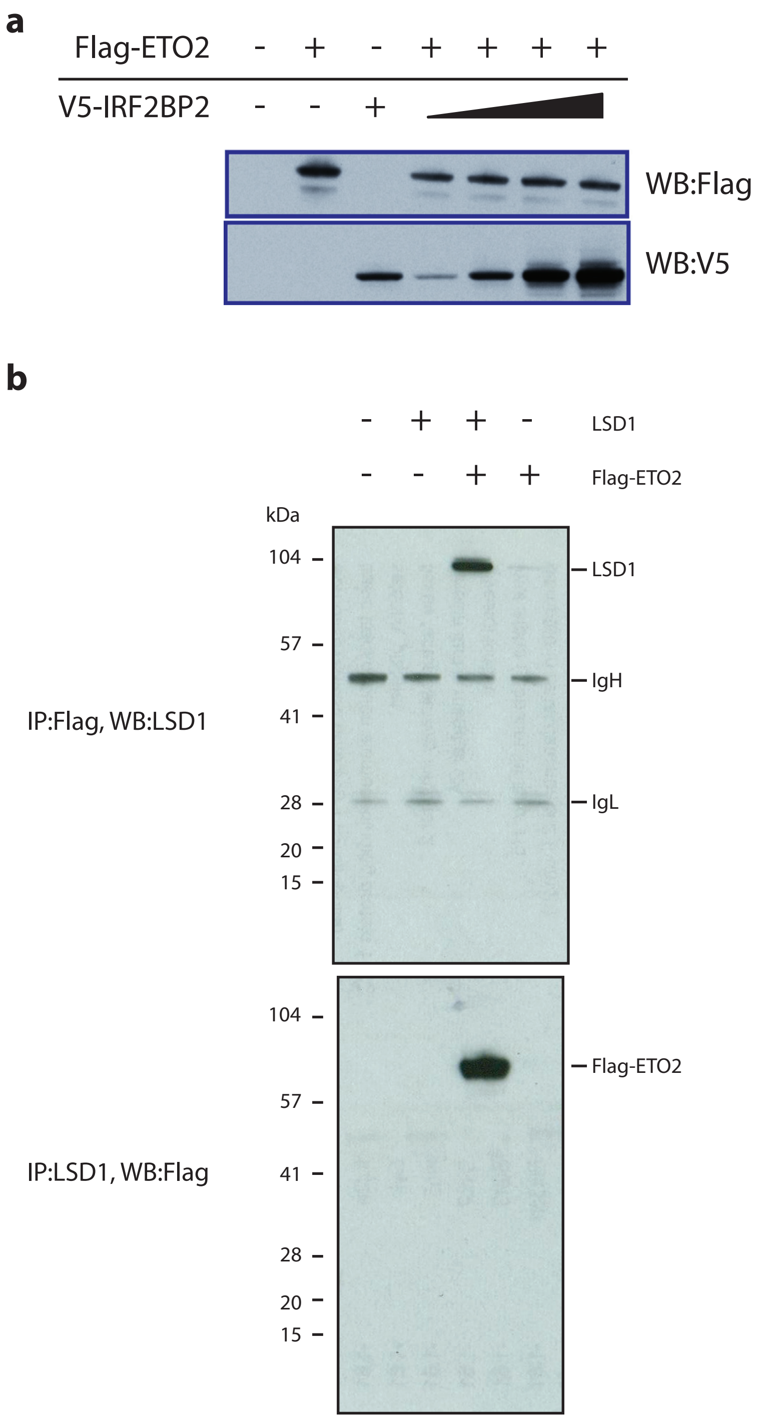


**Supplementary Figure 3. Effect of increasing IRF2BP2 levels on ETO2 protein stability and ETO2-LSD1 co-immunoprecipitation.** (a) Equal amounts of Flag-ETO2 and variable amounts of V5-IRF2BP2 expression constructs were co-transfected into HEK 293T cells. Protein extracts were prepared 48h post-transfection and Flag-ETO2 and V5-IRF2BP2 protein levels were visualized using Western Blot (WB) analysis. Full-size blot images can be found in Supplementary Fig.11. (b) Co-immunoprecipitation experiment showing the interaction of Flag-ETO2 with LSD1 in HEK 293T cells transfected with pcDNA plasmids encoding the two proteins.

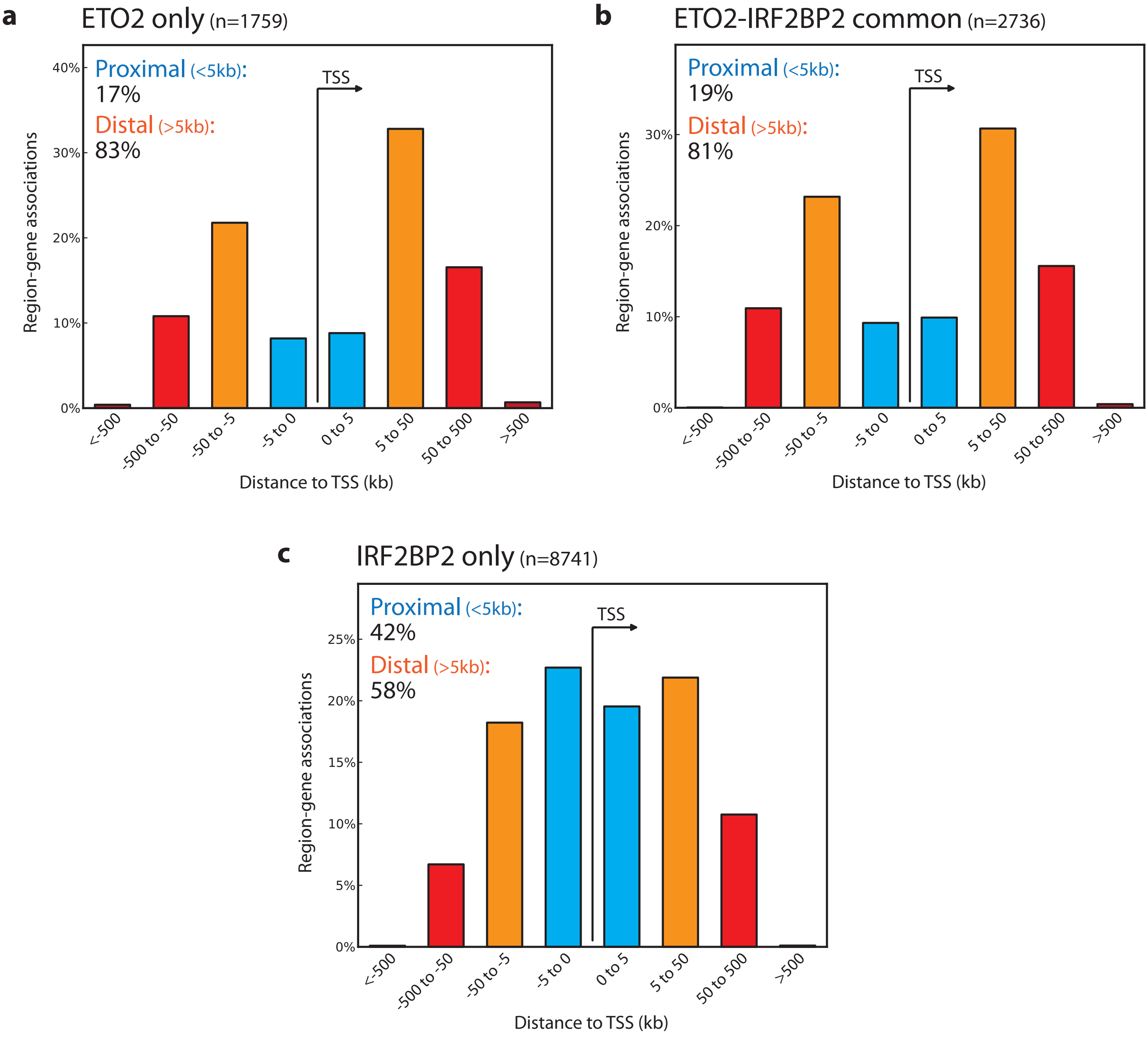


**Supplementary Figure 4. ETO2 and IRF2BP2 ChIP-Seq peak positioning relative to transcription start sites.** The GREAT analysis tool (see Methods) was used to determine the distance of ETO2 only (a), ETO2-IRF2BP2 (b) and IRF2BP2 only (c) binding sites to the nearest transcription start site (TSS). Note the predominantly distal (>80%) binding profile of ETO2 only and ETO2-IRF2BP2 common sites, in accordance with the binding profile of the LDB1-complex (Soler et al. 2010 [ref.6]). In contrast, many genomic sites occupied only by IRF2BP2 (42%) are located in the proximal promoter region.

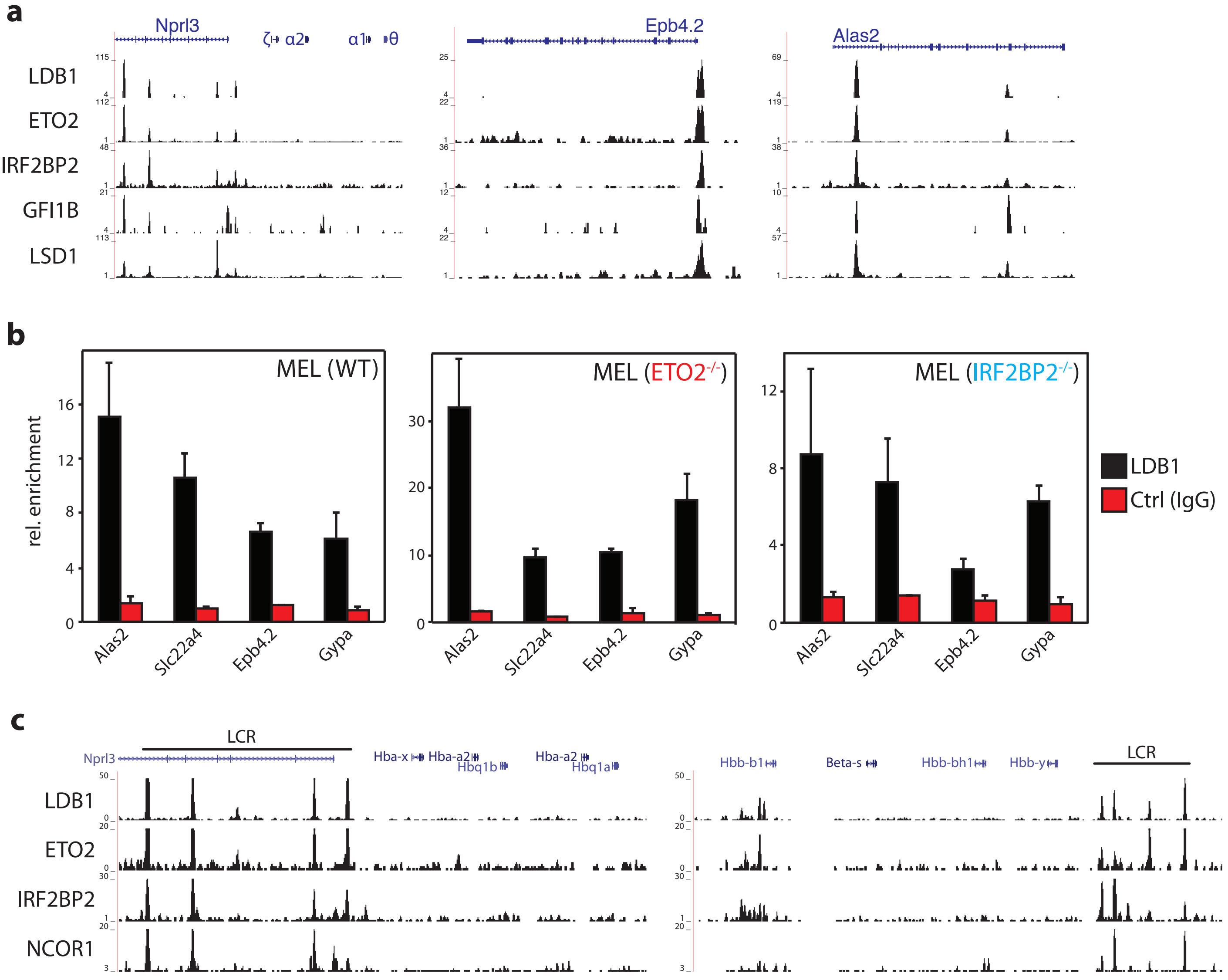


**Supplementary Figure 5. Transcription factor binding profiles and LDB1 ChIP-qPCR in ETO2/IRF2BP2 knockout MEL cell lines.** (a) ChIP-Seq data for LDB1, ETO2, IRF2BP2, GFI1B and LSD1 (from MEL erythroid progenitor cells) is shown for the α-globin (left), *Epb4.2* (middle) and *Alas2* (right) loci. Note the high degree of co-occupancy of the ETO2-interacting corepressor proteins (IRF2BP2, GFI1B and LSD1) on known LDB1/ETO2-complex target genes. (b) ChIP-qPCR experiments showing that LDB1 protein binding in MEL cells is maintained at erythroid genes in the absence of ETO2 or IRF2BP2. Enrichments are shown as relative values over background signal at the amylase gene promoter (n=2-3, error bars denote s.e.m.). (c) ChIP-Seq data for LDB1, ETO2, IRF2BP2, and NCOR1 (from MEL erythroid progenitor cells) is shown for the α-globin (left) and β-globin (right) loci. Note the high degree of co-occupancy of the LDB1 complex (represented here by LDB1, ETO2 and IRF2BP2) and NCOR1 at the locus control region (LCR) that regulates globin gene expression at these loci.

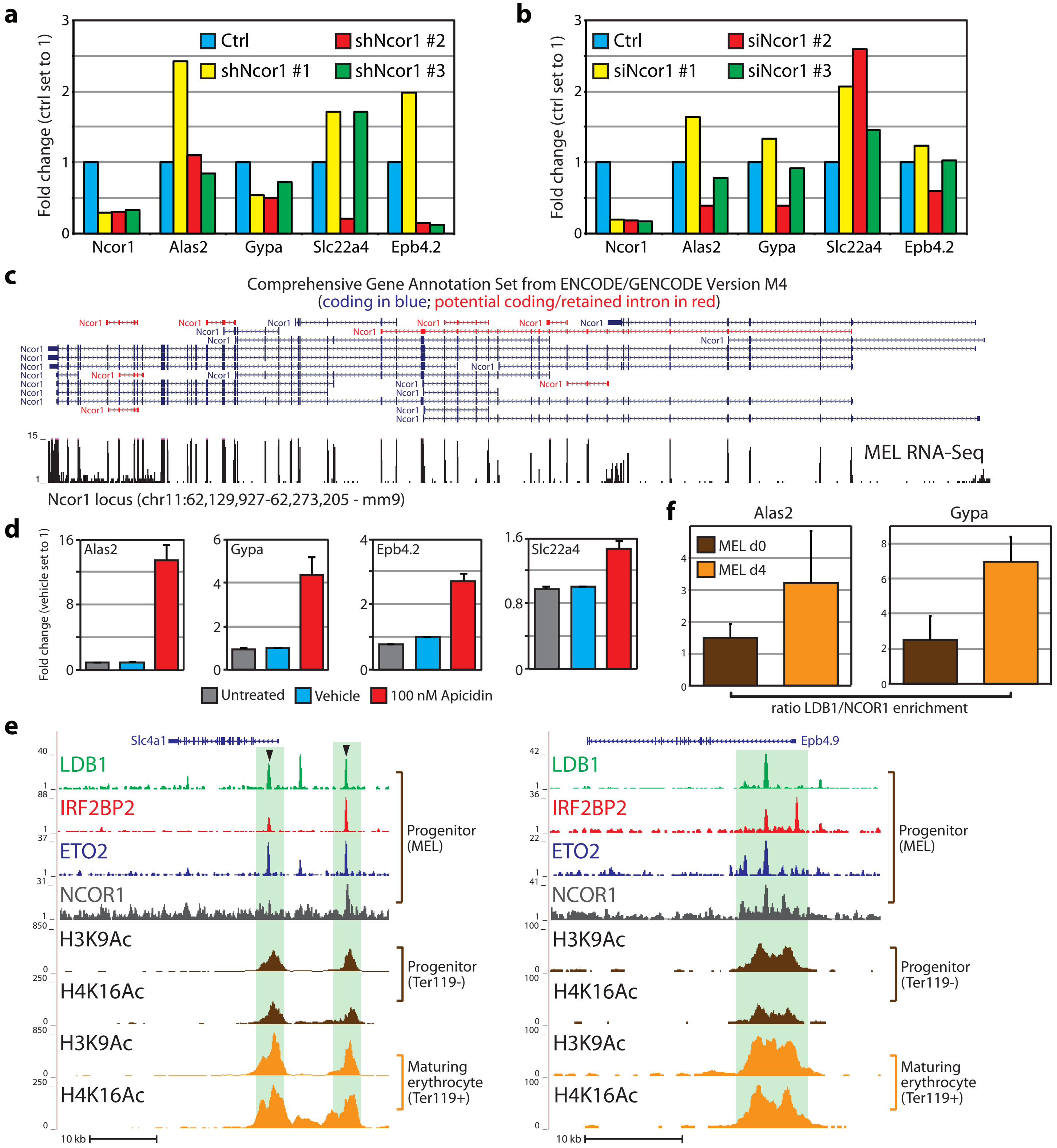


**Supplementary Figure 6. NCOR1 knockdown and HDAC3 inhibition in erythroid progenitors and histone acetylation dynamics at selected primed erythroid genes during development.** (a,b) shRNA (panel a) and siRNA (panel b) mediated knockdown of *Ncor1* mRNA in MEL cells. qPCR gene expression analysis was performed 48-72h after transduction/transfection (normalized versus *Rnh1* levels). 3-4 different shRNAs/siRNAs were used; shown is a representative experiment. (c) UCSC browser screenshot depicting RNA-Seq data from MEL cells for the *Ncor1* locus. Note the large amount of protein-coding isoforms potentially expressed in MEL cells. (d) MEL cells were treated with either vehicle (DMSO) or 100 nM of Apicidin and harvested after 24h for erythroid gene expression analysis by qPCR (normalized versus *Rnh1* levels). Untreated cells were analyzed in parallel as an additional control. Bar graphs depict the average of two independent experiments. Error bars denote s.d. (e) ChIP-Seq profiles for the *Slc4a1* and *Epb4.9* loci showing LDB1, IRF2BP2, ETO2 and NCOR1 co-occupying regulatory elements controlling these two archetypical late erythroid genes in MEL cells. Histone acetylation profiles (H3K9Ac and H4K16Ac, taken from Wong et al. 2010 [ref.21]) from primary erythroid progenitors (Ter119-) and maturing erythrocytes (Ter119+) are shown below. (f) Ratios of LDB1 over NCOR1 ChIP enrichments (n=2, error bars denote s.d.) at selected primed erythroid genes during MEL cell differentiation.

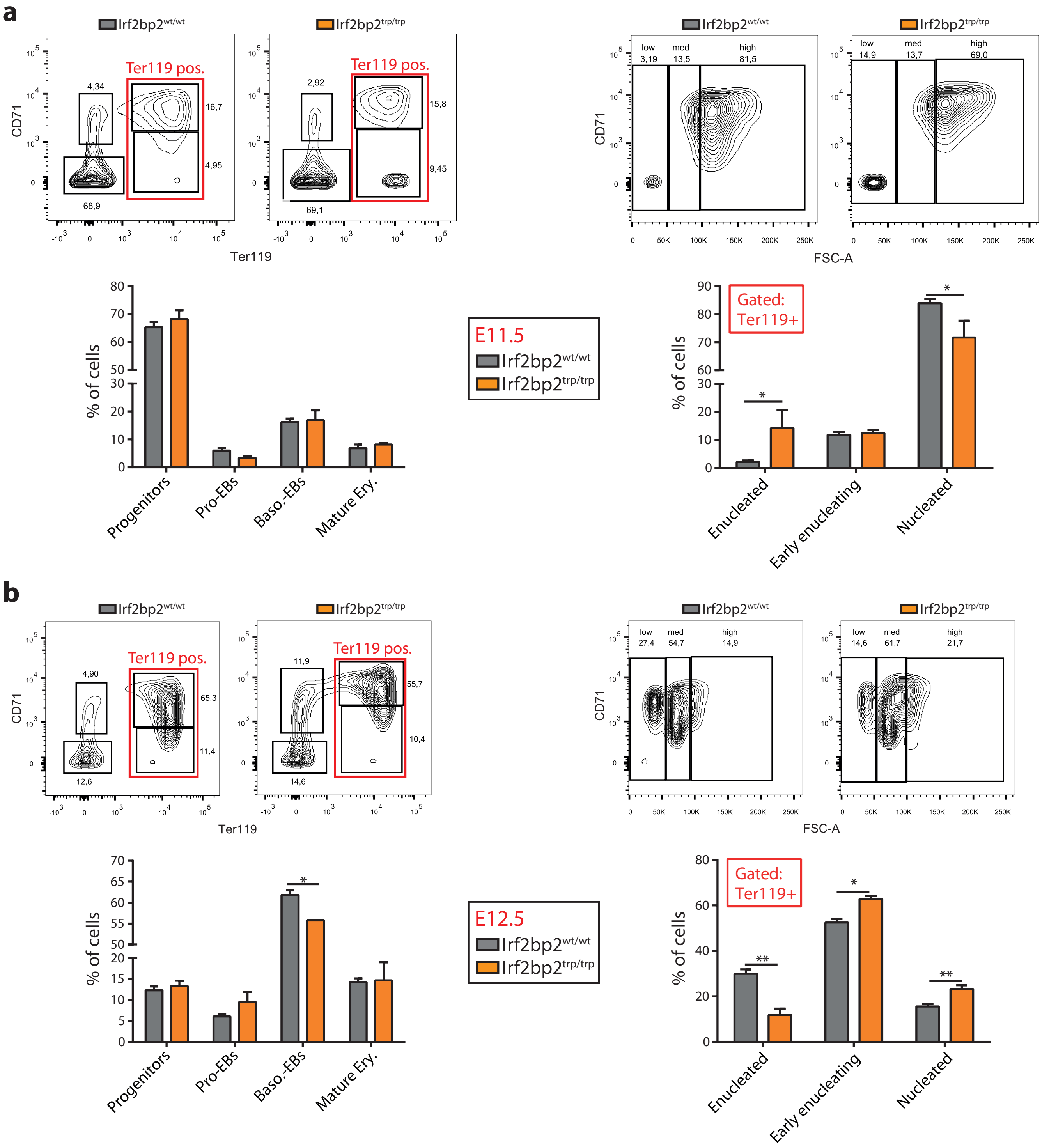


**Supplementary Figure 7. Flowcytometry analysis of E11.5 and E12.5 fetal liver erythropoiesis in IRF2BP2-deficient mice.** Flowcytometry analysis (CD71-Ter119 double staining) of fetal livers from E11.5 (a) or E12.5 (b) embryos with the indicated genotypes (n=3-4 embryos per genotype). Representative flowcytometry plots are shown on top; average values are plotted as bar graphs underneath. The right side of the figure shows Ter119$^{+}$ FL cells separated into three populations based on FSC profile (as in Figure 7). Differences between wildtype and Irf2bp2$^{trp/trp}$ embryos were tested for statistical significance (Mann Whitney U test; *P<0.05, **P<0.01). Error bars denote s.d.

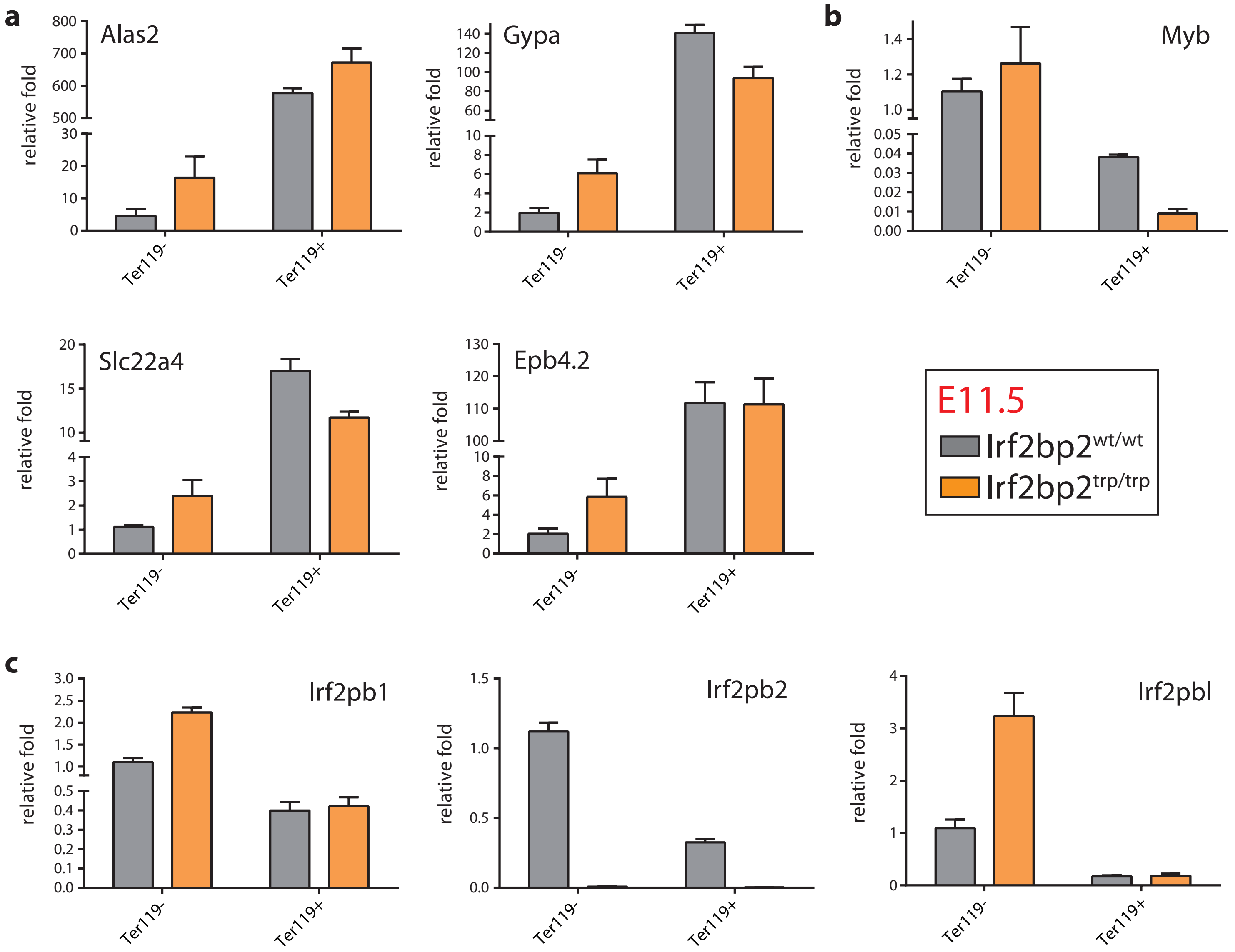


**Supplementary Figure 8. Gene expression analysis on IRF2BP2-deficient erythroid cell populations purified from E11.5 fetal liver.** Gene expression comparison between wildtype and Irf2bp2$^{trp/trp}$ E11.5 FACS-sorted erythroid populations separated into Ter119- erythroid progenitors and Ter119+ maturing erythrocytes. mRNA levels of late erythroid genes (a), the immature marker *Myb* (b) and the three *Irf2bp* family members (c) were measured using qPCR (normalized versus *Rnh1* levels). Bar graphs depict the average of at least two independent experiments. Error bars denote s.d.

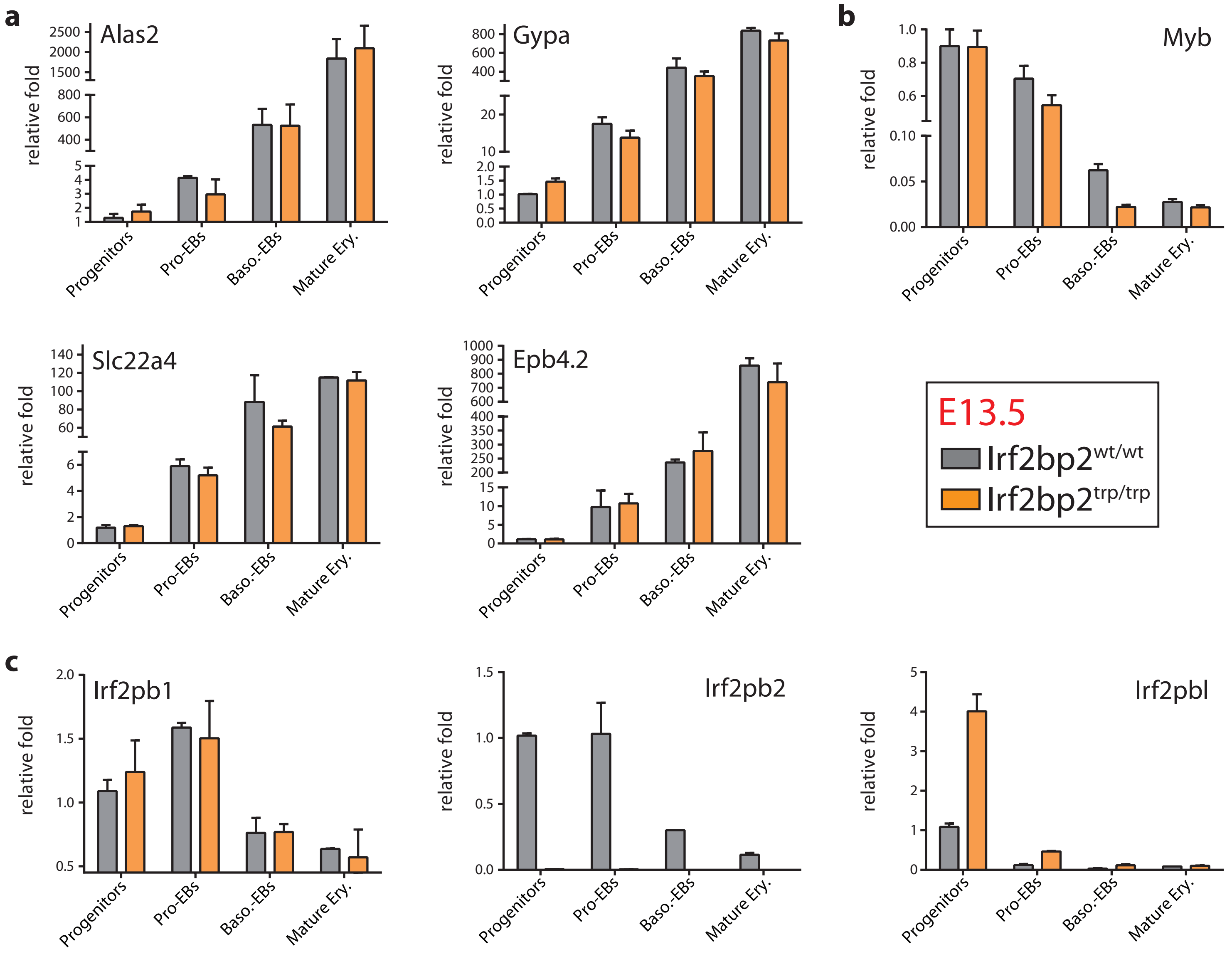


**Supplementary Figure 9. Gene expression analysis on IRF2BP2-deficient erythroid cell populations purified from E13.5 fetal liver.** Gene expression comparison between wildtype and Irf2bp2$^{trp/trp}$ E13.5 FACS-sorted erythroid populations separated into CD71-/Ter119- progenitors, CD71+/Ter119- pro-erythroblasts (pro-EBs), CD71+/Ter119+ basophilic erythroblasts (Baso.-EBs) and CD71-/Ter119+ mature erythrocytes (Mature Ery.). mRNA levels of late erythroid genes (a), the immature marker *Myb* (b) and the three *Irf2bp* family members (c) were measured using qPCR (normalized versus *Rnh1* levels). Bar graphs depict the average of at least two independent experiments. Error bars denote s.d.

## Full scans of blots corresponding to:

**Fig.1a**

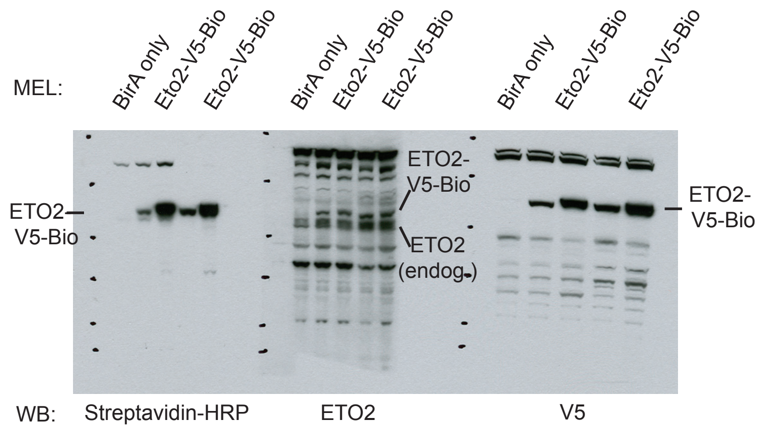


**Fig.1b**

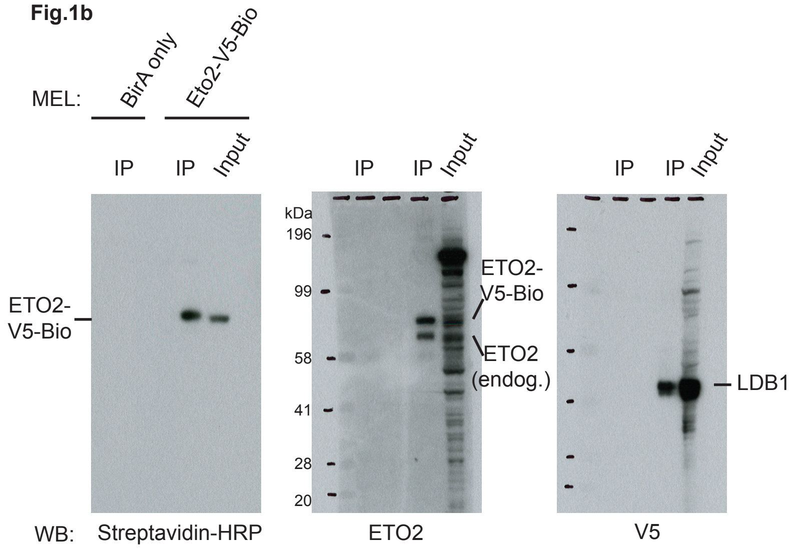


**Fig.1d and Supplementary Fig.2b**

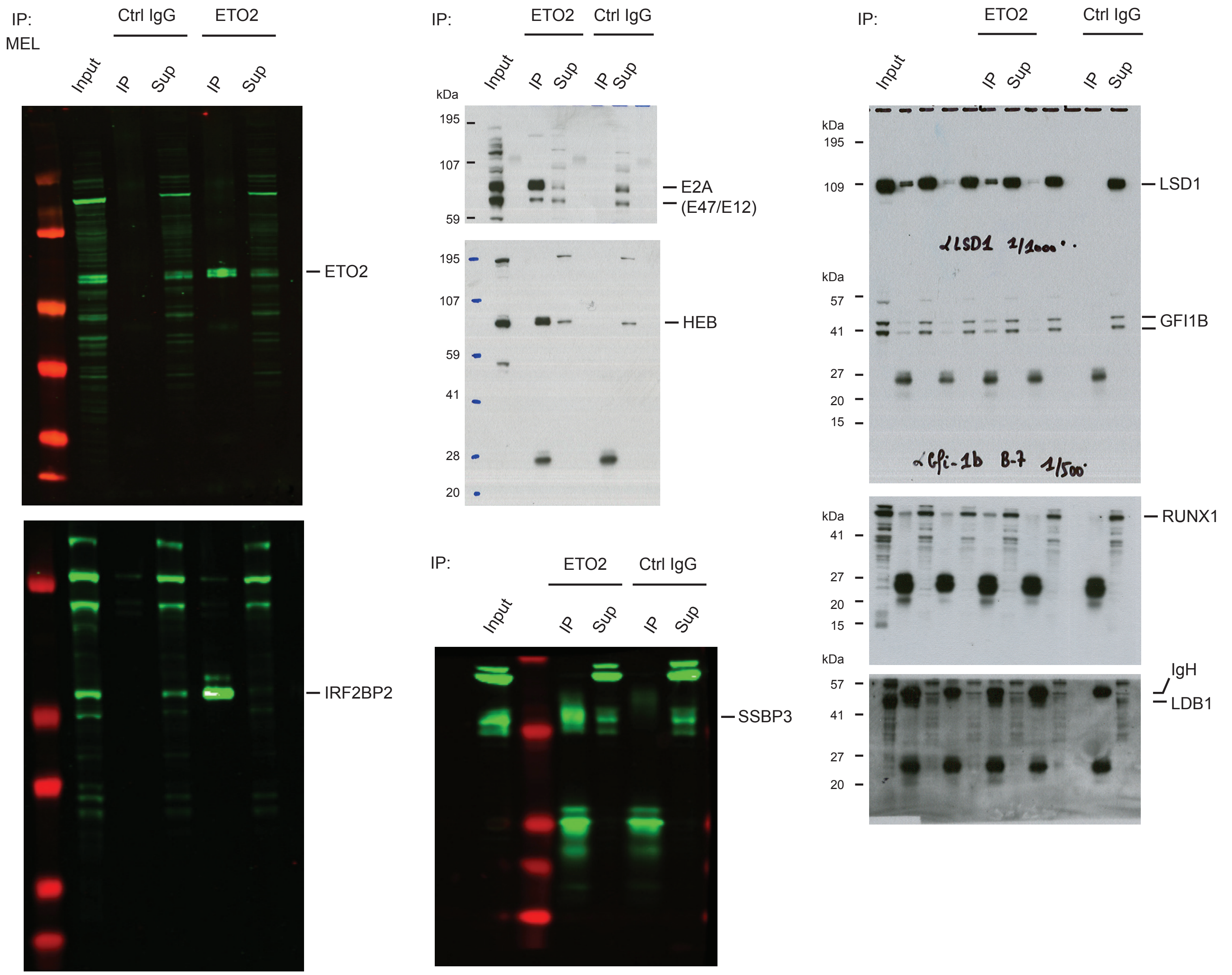


**Supplementary Figure 10. Annotated full image scans of Western blots shown in Fig.1 and Supplementary Fig.2.**

## Full scans of blots corresponding to:

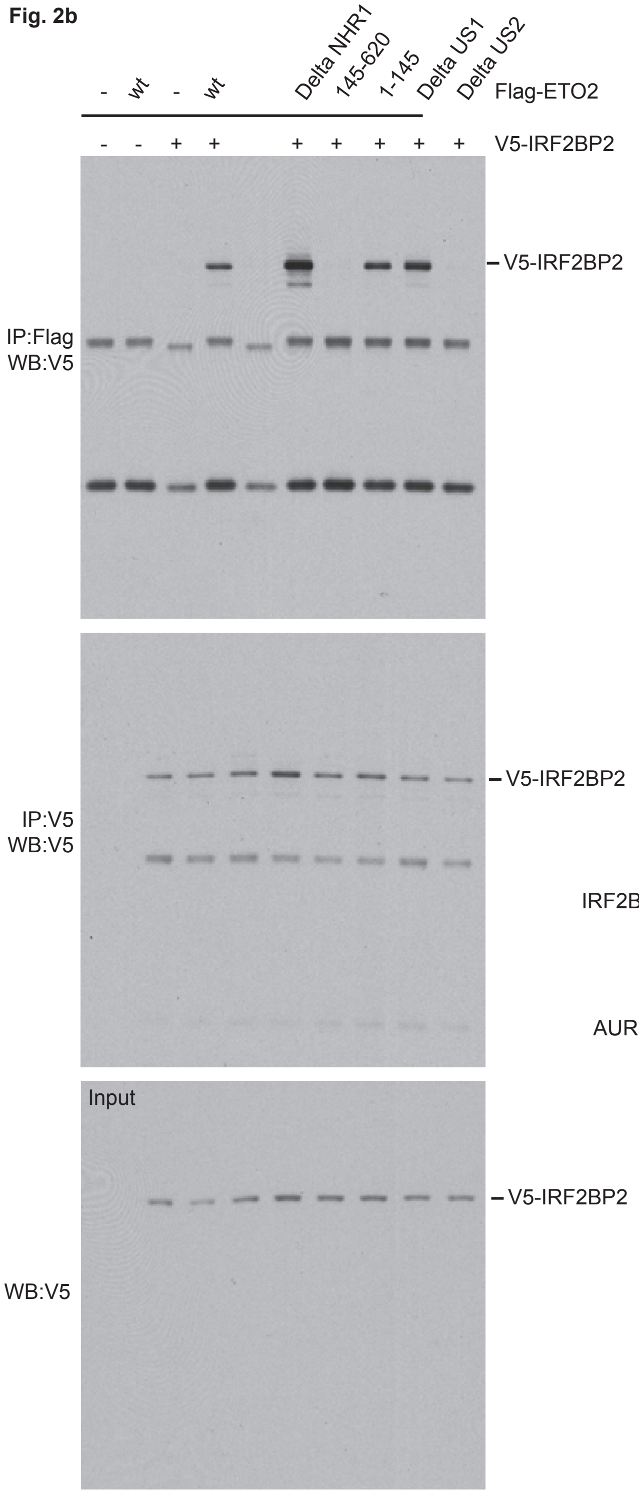


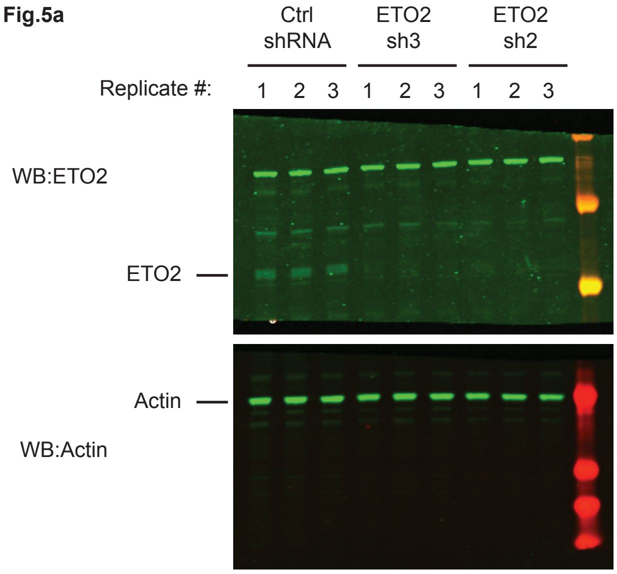


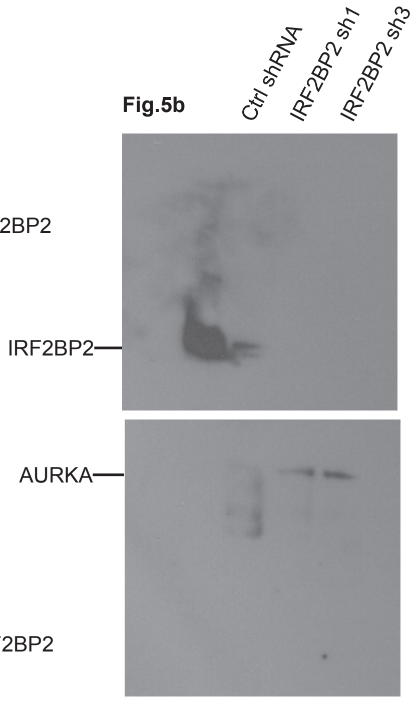


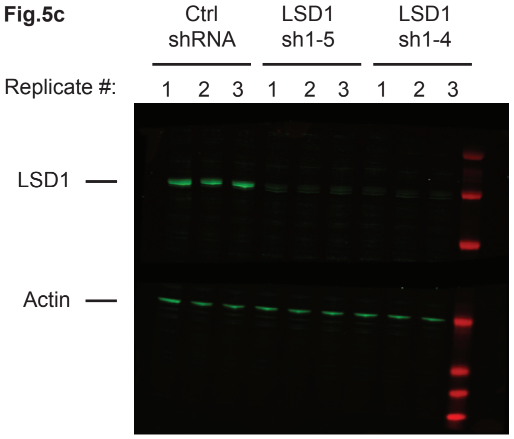


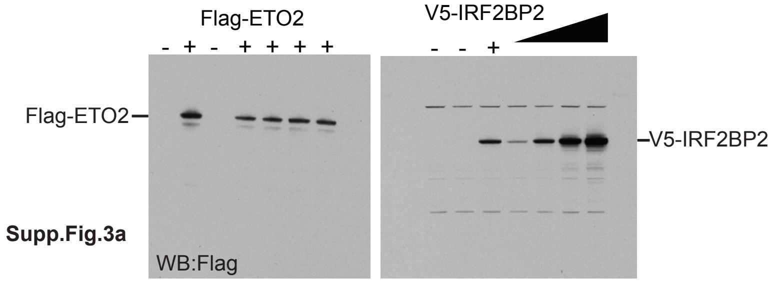


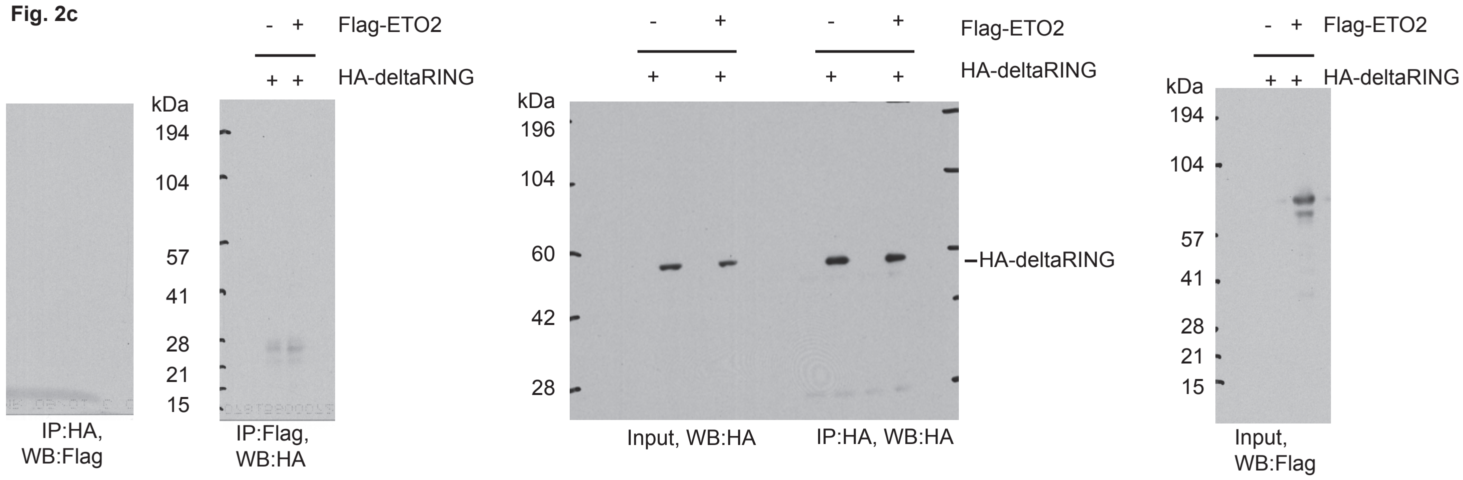


**Supplementary Figure 11. Annotated full image scans of Western blots shown in Fig.2, Fig.5 and Supplementary Fig.3.**

## Supplementary Table 1. Oligonucleotide sequences used in this study.

### Expression Primers

| *Primer name* | *Target mRNA* | *Sequence (5'-3')* |
|---|---|---|
| Alas2-78F | Alas2 | TTAGCCACTTTGCCCAGGAG |
| Alas2-78R | Alas2 | CCGTCTTTGGTTCGTCCTCA |
| Cbfa2t3-F | ETO2 | GTGAACTCAACTCTGACGAT |
| Cbfa2t3-R | ETO2 | GCAGCAGTGGAAGATTAGC |
| Epb4.2-F | Epb4.2 | GCTTTATCTACCTGGGCAC |
| Epb4.2-R | Epb4.2 | TCACTTGCTTGTCCATACTC |
| Gypa-F | Gypa | TGAAGTGTCTGCTGCGTT |
| Gypa-R | Gypa | CCGATAATCCCTGCCATCA |
| Irf2bp1-F | Irf2bp1 | TACCCGGACACAAGTTCTGC |
| Irf2bp1-R | Irf2bp1 | GACAGAGGAACCCACCAGTG |
| Irf2bp2-F | Irf2bp2 | GGCAGGTTGTTGGGTTTC |
| Irf2bp2-R | Irf2bp2 | CAAGGCTGTGTCTCACCA |
| Irf2bpL-F | Irf2bpl | GCGGTTCAAGGGTCTCTCTC |
| Irf2bpL-R | Irf2bpl | TACCTTGGGCCTTCATGCAG |
| Myb-1121-1235-F | Myb | CGACGAAGACCCTGAGAAGG |
| Myb-1121-1235-R | Myb | GCTGCAAGTGTGGTTCTGTG |
| Ncor1-F | Ncor1 | ACTGTGCTGTCTGGTTCC |
| Ncor1-R | Ncor1 | CACCTTCAAATGCTCGGA |
| Slc22a4-F | Slc22a4 | TCACCACCTCCCTGTTCTT |
| Slc22a4-R | Slc22a4 | TTTCTTCCTGCCAAACCTG |
| Slc4a1-F1 | Slc4a1 | GCTCTTCCCACAGAGCAAAC |
| Slc4a1-R1 | Slc4a1 | CTGCCTCCACCCATTGTAGT |
| Rnh1-F | Rnh1 | TGCAGGCACTGAAGCACCA |
| Rnh1-R | Rnh1 | TCCAGTGTGAGCAGCTGAG |

### ChIP Primers

| *Primer name* | *Target genomic region* | *Sequence (5'-3')* |
|---|---|---|
| Alas2-F | *Alas2* intron1 | GGAACTGGGACATCTTGAC |
| Alas2-R | *Alas2* intron1 | ACCATTAGAGTCTGGCTACT |
| Amy-F | Amylase promoter | CTCCTTGTACGGGTTGGT |
| Amy-R | Amylase promoter | AATGATGTGCACAGCTGAA |
| Epb4.2-F | *Epb4.2* promoter | TGAGAAGACTTGCTGGCT |
| Epb4.2-R | *Epb4.2* promoter | GTTCAGAGTTGGGTTCGGA |
| Gata1-enh-F | *Gata1* -3.5kb enhancer | TCAGGGAAGGATCCAAGGAA |
| Gata1-enh-R | *Gata1* -3.5kb enhancer | CCGGGTTGAAGCGTCTTCT |
| Gata1-neg-F | *Gata1* upstream region | CACTAGCAGCTGGGTGGGTTA |
| Gata1-neg-R | *Gata1* upstream region | TGCCGCTTGCCTTTGTAAG |
| Gypa-F | *Gypa* intron3 | TCCTCTCCTTACACCCTGTCT |
| Gypa-R | *Gypa* intron3 | CTCTTACCAACAAGCCAGCC |
| Klf1-enh-F | *Klf1* promoter/enhancer | CTGGCCCCCCTACCTGAT |
| Klf1-enh-R | *Klf1* promoter/enhancer | GGCTCCCTTTCAGGCATTATC |

| | | |
|---|---|---|
| Klf1-neg-F | *Klf1* upstream region | TGCTCCCCACTATGATAATGGA |
| Klf1-neg-R | *Klf1* upstream region | GCCACAACCAAAGAAGACATTTT |
| Slc22a4-F | *Slc22a4* intron1 | TCTGTTGACTGCTCTGTAGT |
| Slc22a4-R | *Slc22a4* intron1 | TAGGTTCCTCCCAATGAGAT |

**Mouse Genotyping Primers**

| *Primer name* | *Target genomic region* | *Sequence (5'-3')* |
|---|---|---|
| V76-F | Gene-trap vector | CTTGCAAAATGGCGTTACTTAAGC |
| Irf2bp2-F2 | *Irf2bp2* gene | GGGTGGGTGGTAGGGATCC |
| Irf2bp2-R2 | *Irf2bp2* gene | GGGCTCTGAGCCTGCACA |

**Crispr gRNA cloning**

| *Primer name* | *Target genomic region* | *Sequence (5'-3')* |
|---|---|---|
| Eto2-N3-F | *Cbfa2t3* intron 5 | CACCGGACTGGGGCCTCACAAACGA |
| Eto2-N3-R | *Cbfa2t3* intron 5 | AAACTCGTTTGTGAGGCCCCAGTCC |
| Eto2-C3-F | *Cbfa2t3* intron 3 | CACCGGAACGGTTGCAGGGACAGAG |
| Eto2-C3-R | *Cbfa2t3* intron 3 | AAACCTCTGTCCCTGCAACCGTTCC |
| IRF2BP2-N3-F | *Irf2bp2* exon 1 | CACCGGGTCAACGGTTCTGCCGCGC |
| IRF2BP2-N3-R | *Irf2bp2* exon 1 | AAACGCGCGGCAGAACCGTTGACCC |
| IRF2BP2-C2-F | *Irf2bp2* exon 2 (3'UTR) | CACCGGGCTTTCCTGCTGACCAGCC |
| IRF2BP2-C2-R | *Irf2bp2* exon 2 (3'UTR) | AAACGGCTGGTCAGCAGGAAAGCCC |

*(for guide RNA primer sequences, the target genomic sequence is underlined)*

**Crispr-deleted MEL KO clones genotyping**

| *Primer name* | *Target genomic region* | *Sequence (5'-3')* |
|---|---|---|
| Eto2-Del-F1 | *Cbfa2t3* intron 5 | AAAGGGCGAGAGCCACAGCTCA |
| Eto2-Del-R | *Cbfa2t3* intron 3 | CAGTCTGGGAGTTTGAACTGGGCAT |
| IRF2BP2-Del-F | *Irf2bp2* exon 2 (3'UTR) | TGCAGGCTTCCTCTACCCACACT |
| IRF2BP2-Del-R | *Irf2bp2* exon 1 | CGCTCAGGCTATGGAGCGCTA |

## Supplementary References


1 Kassouf, M. T. *et al.* Genome-wide identification of TAL1's functional targets: insights into its mechanisms of action in primary erythroid cells. *Genome research* **20**, 1064-1083, doi:10.1101/gr.104935.110 (2010).

2 Soler, E. *et al.* The genome-wide dynamics of the binding of Ldb1 complexes during erythroid differentiation. *Genes & development* **24**, 277-289, doi:10.1101/gad.551810 (2010).

3 Wong, P. *et al.* Gene induction and repression during terminal erythropoiesis are mediated by distinct epigenetic changes. *Blood* **118**, e128-138, doi:10.1182/blood-2011-03-341404 (2011).
